# HIPSD&R-seq enables scalable genomic copy number and transcriptome profiling

**DOI:** 10.1186/s13059-024-03450-0

**Published:** 2024-12-18

**Authors:** Jan Otoničar, Olga Lazareva, Jan-Philipp Mallm, Milena Simovic-Lorenz, George Philippos, Pooja Sant, Urja Parekh, Linda Hammann, Albert Li, Umut Yildiz, Mikael Marttinen, Judith Zaugg, Kyung Min Noh, Oliver Stegle, Aurélie Ernst

**Affiliations:** 1 Group Genome Instability in Tumors, German Cancer Research Center (DKFZ), Heidelberg, Germany; 2 German Cancer Consortium (DKTK), DKFZ, Core Center, Heidelberg, Germany; 3 Faculty of Biosciences, Heidelberg University, Heidelberg, Germany; 4 Division of Computational Genomics and Systems Genetics, German Cancer Research Center, Heidelberg, Germany; 5 European Molecular Biology Laboratory, Genome Biology Unit, Heidelberg, Germany; 6 Junior Clinical Cooperation Unit, Multiparametric Methods for Early Detection of Prostate Cancer, German Cancer Research Center (DKFZ), Heidelberg, Germany; 7 Single Cell Open Lab, German Cancer Research Center (DKFZ), Heidelberg, Germany; 8 Center for Quantitative Analysis of Molecular and Cellular Biosystems (BioQuant), Heidelberg University, Heidelberg, Germany; 9 European Molecular Biology Laboratory, Structural and Computational Biology Unit, Heidelberg, Germany; 10 Molecular Medicine Partnership Unit, University of Heidelberg, Heidelberg, Germany; 11 Wellcome Sanger Institute, Wellcome Genome Campus, Cambridge, UK

**Keywords:** Single Cell DNA sequencing, Single Cell multiome, Single Cell copy Number profiling

## Abstract

Single-cell DNA sequencing (scDNA-seq) enables decoding somatic cancer variation. Existing methods are hampered by low throughput or cannot be combined with transcriptome sequencing in the same cell. We propose HIPSD&R-seq (HIgh-throughPut Single-cell Dna and Rna-seq), a scalable yet simple and accessible assay to profile low-coverage DNA and RNA in thousands of cells in parallel. Our approach builds on a modification of the 10X Genomics platform for scATAC and multiome profiling. In applications to human cell models and primary tissue, we demonstrate the feasibility to detect rare clones and we combine the assay with combinatorial indexing to profile over 17,000 cells.

**Supplementary Information:**

The online version contains supplementary material available at 10.1186/s13059-024-03450-0.

## Background

Advances in single-cell DNA sequencing (scDNA-seq) technologies have enabled exploring new dimensions of genomic diversity that are not accessible by bulk sequencing. scDNA-seq methods have been instrumental in the fields of somatic mutation and mosaicism [1–6], organ development [7–11], germ cell mutation and fertility [12–16], epigenetic regulation and genome organization [17–23], and cancer research [1, 2, 24–27]. Established protocols can be broadly categorized into targeted sequencing methods, i.e., focus on individual mutations of interest [28–34], or are based on low-coverage genome-wide sequencing for the identification of copy number variants or somatic alterations (CNVs, where the number of copies varies between individuals, and CNAs, respectively) [35–39]. Here, we focus on the latter class of methods, which have been successfully applied to unravel clonal composition in oncology, with implications in precision oncology and resistance to cancer treatment [40, 41].

Early scDNA-seq protocols have been extended and improved to increase coverage and reduce bias (the latter through amplification-free workflows for example) [26, 42–44], and to allow for parallel profiling of DNA and RNA from the same cells [36, 37, 42–44]. Most recently, with the advent of combinatorial indexing, the feasibility of scaling scDNA-seq to tens of thousands of cells in a single experiment has been demonstrated [48]. However, to date there is a lack of schemes that render high-throughput scDNA-seq accessible to the community and reproducible across laboratories. Commercially available technologies such as the 10 × platforms have had tremendous impact on RNA and epigenome profiling, yet there is no equivalent for the scalable profiling of genomic variation in single cells. In fact, the lack of accessible scDNA-seq protocols has stimulated the development of computational solutions to infer coarse grained CNAs from ATAC and RNA abundance profiles, a strategy that is recognized to have lower resolution and accuracy [49, 50], yet achievable using commercial single-cell ATAC or RNA sequencing (scATAC-seq and scRNA-seq) platforms [49, 51–53].

Key challenges of current protocols for scDNA-seq [2, 26, 42–47, 54–58] include low throughput and/or difficulty to establish these methods elsewhere, for instance due to the need for specialized equipment [59]. As compared to scRNA-seq, which has been democratized by commercially available systems [60–69], scDNA-seq and parallel single-cell DNA and RNA sequencing (scDNA/RNA-seq) are lagging behind. For parallel analyses of copy-number variation and transcriptomes from the same cells, pioneering studies have focused on low to medium throughput methods like DR-Seq (13 to 33 cells) [42], Simul-seq (8 to 10 cells) [70], scTrio-seq (25 cells) [37], SIDR-seq (74 cells) [46], scONE-seq (86 cells) [71], G&T-seq (220 cells), or DNTR-seq (230 cells) [72], which have been applied to at most hundreds of cells. sci-L3 recently provided a proof-of principle in cell lines for the analysis of DNA and RNA of thousands of cells [73], with scalable combinatorial indexing as a major advance. A notable exception is the approach proposed developed in parallel by Peter Sims and colleagues [74], which shares some of the characteristics of our method. However, our approach and in particular the DNA-component provides several advantages, and we achieve an order of magnitude more throughput by combining the assays with combinatorial indexing.

Here, we propose HIgh-throughPut Single-cell Dna and Rna-seq (HIPSD&R-seq), a scalable yet simple assay, which offers high-throughput scDNA-seq from several hundreds to tens of thousands of cells, coupled with the benefits of an established workflow that builds on the commercial 10 × Genomics platform. We compare HIPSD&R-seq to alternative scDNA-seq protocols, to scATAC-seq and to CNV inference based on scRNA-seq, finding substantial benefits in resolution and robustness. Finally, we demonstrate the utility of the throughput provided by HIPSD&R-seq in the context of a spike-in experiment to detect rare clones, and we apply the assay to human fibroblasts and to medulloblastoma patient-derived xenografts.

## Results

**Fig. 1:**
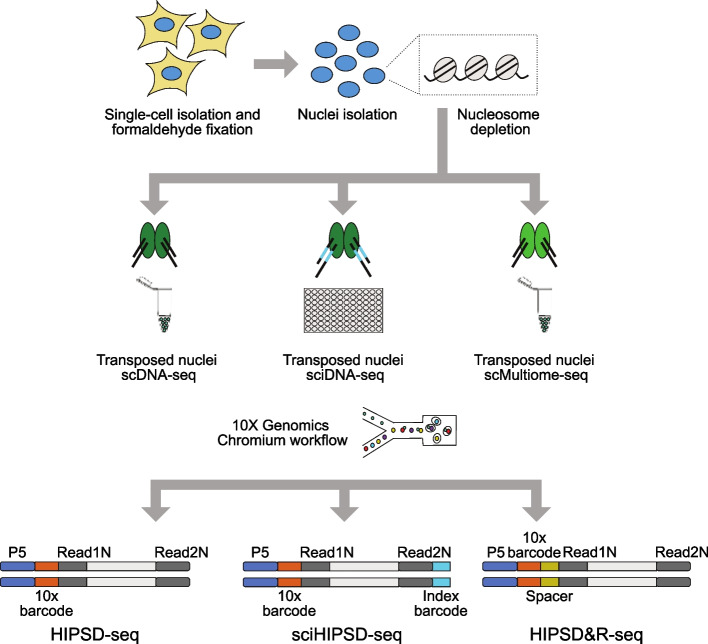
Schematic representation of the experimental approach for three connected protocols based on a modular modified 10 × Genomics workflow—HIPSD-seq, sci-HIPSD-seq, and HIPSD&R-seq. Formaldehyde fixed single-cell suspensions are used for nuclei isolation, followed by SDS-based nucleosome depletion. The nucleosome-depleted genomic regions are randomly tagmented using the transposase Tn5 (HIPSD-seq and HIPSD&R-seq) or barcoded Tn5 (sci-HIPSD-seq). Transposed nuclei are processed using the 10 × Genomics Chromium single cell ATAC workflow (HIPSD-seq and sci-HIPSD-seq) or the multiome workflow (HIPSD&R-seq) to generate single-cell DNA and gene expression (RNA) libraries for sequencing. Combining scDNA-seq with an additional combinatorial indexing step thanks to the barcoded Tn5 leads to an ultra-high throughput (sci-HIPSD-seq)

We have developed HIPSD&R-seq, a modular experimental workflow that allows for implementing scalable single-cell DNA and optionally RNA sequencing from the same cells (Fig. 1). We will use the words nuclei and cells interchangeably. Core to our approach is the repurposing of existing scATAC-seq and multiome protocols to perform high-throughput scDNA-seq (Fig. 1). Briefly, Tn5 has been a versatile tool for many sequencing applications, including ATAC-seq [18], RNA library preparation [75–77], 3D chromatin structure [78–81], and also DNA-seq [36, 42, 46]. We utilized highly concentrated Tn5 in situ by fixing cells mildly with formaldehyde, depleting nucleosomes with SDS followed by the transposition reaction in still intact nuclei. Combined with the 10X Genomics scATAC-seq kit to encapsulate cells, this modification provides the means to achieve medium-to-high-throughput single-cell DNA-seq (HIPSD-seq, > 5000 cells per sample). The highly concentrated Tn5 replaced the supplied enzyme by 10X to ensure high integration rates across the genome [82]. The same modification of the genome tagmentation can also be combined with the 10X multiome kit to enable parallel scDNA-seq and RNA sequencing (HIPSD&R-seq). Notably, HIPSD-seq can also be combined with an additional combinatorial indexing step [48] to achieve ultra-high throughput scDNA-seq (sci-HIPSD-seq, > 17,000 cells) (“Methods”). Data from all three protocols can be processed using the standard Cell Ranger pipelines with adjustments for the cell calling (Additional file 1: Figs. S1–2, “ Methods”). We have further developed computational strategies to aggregate read data across cells, by adapting existing metacelling strategies from scRNA-seq [83–85] to scDNA-seq (“ Methods”). By aggregating reads data from genetically similar cells, this approach greatly improves read coverage and uniformity while preserving rare clones.

First, to assess the quality of HIPSD- and HIPSD&R-seq libraries, we processed nuclei from human fibroblasts with high genome instability [86]. HIPSD-seq and HIPSD&R-seq yielded more than 5000 high-quality cells (Additional file 2: Table S1, Additional file 1: Figs. S1–3). In comparison to DEFND applied to the same source sample, we showed lower RNA contamination, better separation of patients in a mixed patient experiment, and a higher correlation with CNV profiles derived from matching bulk WGS of the source samples (Additional file 1: Figs. S1–2). We assessed canonical quality control metrics, including read uniformity and the total DNA sequence coverage (Additional file 1: Fig. S3). While HIPSD-seq on individual cells yields lower coverage than existing scDNA-seq methods, the combination of HIPSD-seq with metacelling yields data that are comparable to existing sparse scDNA-seq methods (such as DEFND and the Chromium Single Cell CNV assay, see Additional file 1: Fig. S3). As a reference for desired quality characteristics, we compared quality control metrics of HIPSD-seq data to that from the discontinued Chromium Single Cell CNV assay [87], obtained from the same PDX sample. While the coverage is lower in HIPSD-seq as compared to the Chromium Single Cell CNV assay, the throughput is ~ 10-times higher (Additional file 1: Figs. S3–4, “Methods”, for more details see also Smirnov et al. [88]). Additionally, we compared the quality of transcriptome data obtained from HIPSD&R-seq to Chromium Single Cell Gene Expression assay applied to the same sample, showing a comparable number of genes and counts per cell when accounting for library size (for more details see also Smirnov et al. [88], Additional file 1: Figs. S3–4, and Additional file 2: Table S1). Finally, we compared libraries generated from HIPSD&R-seq to a standard ATAC-seq library to evaluate the nucleosome depletion efficiency of HIPSD&R-seq and to compare HIPSD&R-seq CNV profiles with scATAC-based CNV inference (Additional file 1: Fig. S5). Libraries from the ATAC-seq protocol showed the characteristic periodicity of insert length derived from nucleosomes and a high TSS enrichment score, as expected (Additional file 1: Fig. S5A–B). In contrast, such a nucleosome-dependent periodicity in fragment length and high TSS enrichment score was not observed in libraries from HIPSD&R-seq or sci-HIPSD-seq (Additional file 1: Fig. S5A–B), indicating that no major ATAC-dependent bias remains after nucleosome depletion. Furthermore, CNV profiles obtained from HIPSD&R-seq showed a higher correlation to bulk-derived CNVs as compared to scATAC-based CNV inference (using epiAneufinder [89]; Additional file 1: Fig. S5C–F).

**Fig. 2:**
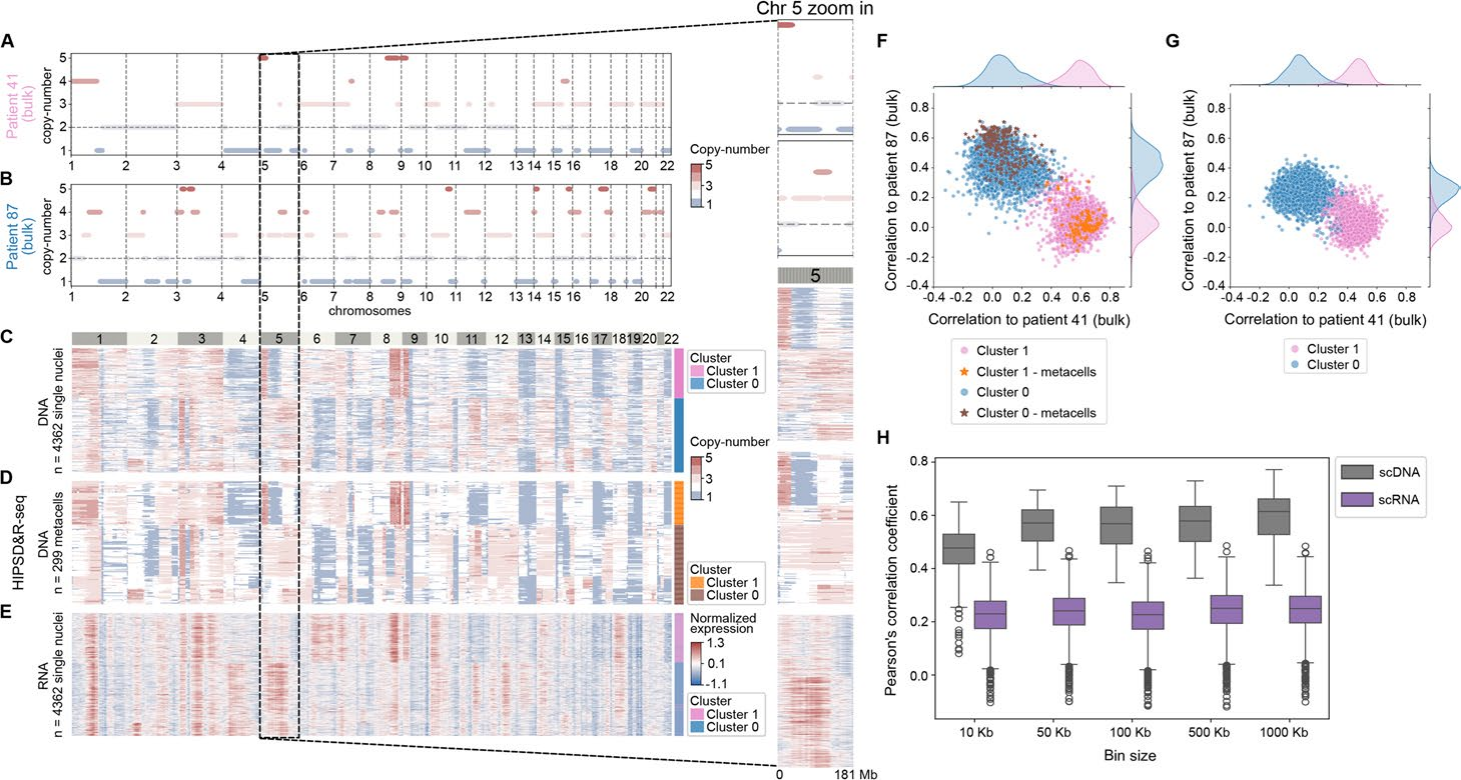
HIPSD&R-seq shows close correspondence to the reference bulk data (1:1 mix of fibroblasts from patient 41 and patient 87). **A** CNV estimates derived from bulk WGS data for patient 41. **B** CNV estimates derived from bulk WGS data for patient 87. **C** CNV estimates derived from HIPSD&R-seq (DNA component) in 4362 single nuclei. Coloring of the clusters is based on **F**. DNA-based CNVs estimated using HMMcopy (1 MB bin size). **D** CNV estimates derived from HIPSD&R-seq (DNA component) in metacells (299 metacells, 100 kb bin size). Coloring of the clusters is based on **F**. **E** CNV estimates derived from the HIPSD&R-seq RNA component (Numbat window-smoothed expression). Cells are ordered as in **C**, but annotated based on unsupervised clustering of CNVs estimated from RNA. **F** Scatter plot of genome-wide Pearson’s correlation coefficients of CNV profiles of individual cells (as in **C**, 1 MB bin size) and metacells (as in **D**, 100 kb bin size) with CNV profiles from bulk WGS of the two source samples (patient 41 and patient 87). The two patients can be robustly identified by unsupervised clustering of CNVs from single nuclei (each blue or pink dot shows one cell) or metacells (each brown or orange dot shows one metacell). Only genomic windows that overlap with those from scRNA in **G** were used to calculate correlations. **G** Analogous correlation coefficients as in **F**, however considering CNV estimates from scRNA-seq. **H** Consistency analysis of CNV estimates from bulk DNA and from the DNA and RNA components of HIPSD&R-seq. Shown are Pearson’s correlation coefficients for pairs of alternative methods, for different bin sizes (10 kb, 50 kb, 100 kb, 500 kb, and 1 MB). Patient 87 and all the cells from RNA component from cluster 0 and metacells from DNA component from cluster 0 were used for this analysis. For each assay, the maximum number of genomic bins overlapping with bulk WGS data was used to calculate correlations. While the Pearson’s correlation coefficient stays reasonably high at 10 kb and 50 kb bin sizes, our metacelling workflow was designed specifically for bin sizes down to 100 kb (see “ Methods”)

After verifying these quality control metrics across samples, we next focused our analysis on HIPSD&R-seq data for low-coverage DNA and RNA sequencing of human fibroblasts from two patients (one male and one female patient) mixed at a 1:1 ratio (Fig. 2, Additional file 2: Table S1), in addition to the patient-derived xenograft of a medulloblastoma with high genome instability used for the benchmarking and mentioned earlier (Additional file 1: Fig. S4). The RNA component of HIPSD&R-seq recovered a median number of over 1800 genes per cell (Table S1), which is in line with data from conventional multiome assays [36, 46]. Motivated by the uptake of computational approaches to infer CNAs from scRNA-seq data, we compared CNV estimates from the DNA component of HIPSD&R-seq to results obtained using inference from the corresponding RNA profiles (using Numbat, “ Methods”) [49]. Qualitatively, the CNV states derived from the DNA component of HIPSD&R-seq (single cells or metacells) offered markedly higher resolution (Fig. 2A–E, Additional file 1: Fig. S4). We also assessed the concordance of the CNV estimates with the corresponding bulk whole-genome CNV profiles from the mixed sample (Fig. 2F–H). This comparison revealed substantially higher consistency for HIPSD-seq compared to CNV inference from the matched RNA (Fig. 2H, Additional file 1: Fig. S6), also in the regime of smaller bin size (down to 100 kb). Although we do not see any major drop in the correlation of CNV estimates between HIPSD-seq and bulk WGS profile for bin sizes 10 kb and 50 kb, the metacelling workflow was specifically designed for applications down to 100 kb bin size (Fig. 2H, Additional file 1: Fig. S6). Overall, HIPSD&R-seq resolves clonal heterogeneity that results from CNVs and provides matched transcriptome profiles, with a higher throughput and lower ambient RNA contamination as compared to the related DEFND protocol [74] (Additional file 1: Figs. S1–2). We also used the HIPSD&R-seq data of the 1:1 mix of fibroblasts from a male and a female patient to identify differentially expressed genes between both patients. This analysis showed that sex-specific genes (e.g., *XIST*) as well as genes located in chromosome regions for which the copy number differs between both patients belong to the most differentially expressed genes (Additional file 1: Fig. S7).

**Fig. 3:**
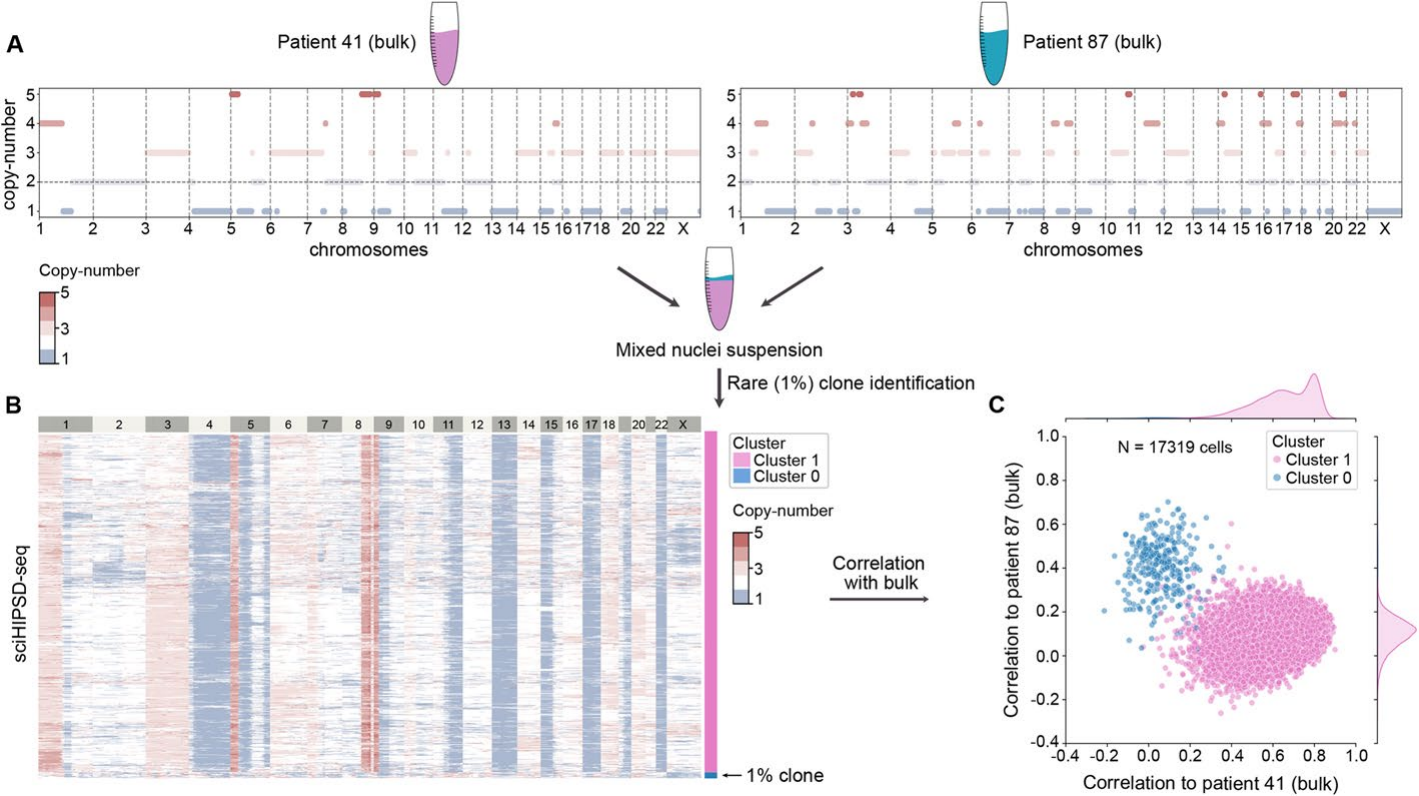
sci-HIPSD-seq identifies a rare clone. **A** Bulk profiles for patient 41 (99% of cells) and patient 87 (1% spike-in). **B** The mixed suspension with nuclei from both patients was analyzed using sci-HIPSD-seq. DNA-based CNVs estimated using HMMcopy (bin size: 1 MB) and the 1% clone can be robustly identified by correlating CNVs from every cell to the two bulk profiles. **C** Correlation to the bulk profiles. The clusters were identified with Leiden clustering of single-cell CNV profiles. *N* = 17,319 cells

Finally, we set out to test ultra-high throughput scDNA-seq using sci-HIPSD-seq, which is particularly suitable to identify rare subpopulations of cells. We performed a spike-in experiment by mixing 1% of nuclei from fibroblasts from a male donor (patient 87) to a nuclei suspension from a female individual (patient 41). The CNV profiles from the combined sample (17,319 single cells after filtering) revealed a subpopulation that is consistent in frequency with the 1% prevalence of the spike-in sample (Fig. 3, Additional file 1: Fig. S8). Assessment of the concordance with the bulk profiles from either patient samples (patient 87 and patient 41) confirmed the identity of both subpopulations, thus demonstrating the ability to detect rare cell populations using sci-HIPSD-seq. As our HIPSD-seq methods are easy, high-throughput, and affordable, they pave the way to applications in cancer genomics, especially in the context of chromosome instability and treatment resistance.

## Discussion

We have established HIPSD&R-seq, a new single-cell DNA sequencing assay, which (i) allows copy-number profiling in thousands of cells, (ii) is compatible with multiome read-out, and (iii) offers sparse but uniform coverage (Additional file 1: Fig. S3). Of note, we have not reached saturation for most experiments; hence, sparse coverage is due to the high throughput rather than the methodology itself. We provide computational workflows that are tailored to analyze sparse data, borrowing concepts from metacelling that were established for scRNA-seq, as well as single-cell analysis workflows [83, 90]. HIPSD&R-seq can be applied to a range of sample types, is easily implemented, and thus widely applicable. We showed that HIPSD&R-seq provides a significant improvement in CNV robustness and resolution compared to inference-based methods applied to the RNA component of the same cells or to ATAC data, and yields results that are markedly concordant with CNVs detected in matched bulk WGS samples. Furthermore, our method works without the need for a reference, which in contrast, plays an important role in CNV inference from scRNA-seq [91]. When combining HIPSD-seq with previously proposed combinatorial indexing to increase the throughput further, we improve the coverage and reach an ultra-high throughput, recovering a subpopulation of cells making up 1% of the total cell population and identifying major clones with distinct CNV profiles.

For the multiome assay, one limitation of the method is the high duplication rate, which is due to (i) ligation of indices and (ii) purification of non-amplified material, as compared to PCR used for barcoding and pre-amplification. In comparison to DEFND, which shows a better coverage and a lower duplication rate, we showed lower RNA contamination, better separation of patients in a mixed patient experiment, and a higher correlation with CNV profiles derived from matching bulk WGS of the source samples. Thus, despite the mentioned limitation, HIPSD&R-seq provides CNV profiles at high resolution and matched transcriptomes for the same cells, opening up a number of applications in cancer research and beyond. As compared to methods such as DLP + , which provide excellent CNV read-outs but require specialized equipment available to very few laboratories in the world, HIPSD-seq can be implemented anywhere. The high yield will lead to a better understanding of genetic heterogeneity and chromosome instability in space and time, by enabling high-throughput analyses of longitudinal and multi-region sampling.

## Conclusions

Dissecting matched single-cell genomes and transcriptomes will lead to a better understanding of the transcriptional consequences of genetic variation and allow to disentangle the links between genome and transcriptome. Ultimately, capturing CNV-based cell lineage trees with cell type and state annotations will help to decipher the role of cell heterogeneity in disease and evolution.

## Methods

### Tn5 loading

Unless otherwise specified we used a high-activity Tn5 transposase [82] and not the 10 × multiome kit Tn5 transposase. Lyophilized adapter oligos were resuspended at 100 μM in annealing buffer (50 mM NaCl, 40 mM Tris pH 8). Adapters (oligo list, see Table S2) were pre-annealed on a thermocycler for 2 min at 85 °C followed by cooling down to 20 °C with 1 °C per minute. For HIPSD-seq, Read1 was annealed with blocked-phos-ME and Read2 with blocked-phos-ME. For HIPSD&R-seq and DEFND-seq with mixed patient fibroblasts, phos-Read1 was annealed with phos-ME and Read2 was annealed with phos-ME. For sci-HIPSD-seq, Read1 was annealed with unblocked-phos-ME and barcoded-Read2 with unblocked-phos-ME. Equal volume of 100% glycerol was then added to the adapters and they were stored at − 20 °C. For Tn5 assembly, transposase and annealed primers are mixed with equal volumes and incubated for 30 min at room temperature. The Tn5 was then diluted in dilution buffer (50 mM Tris pH 7.5, 100 mM NaCl, 0.1 mM EDTA, 1 mM DTT, 0.1% NP-40, 50% glycerol) to 83 µg/mL for HIPSD-seq.

### Cell culture and nuclei extraction from fibroblasts

#### Sample preparation for fibroblasts with high genome instability

Fibroblasts from patients with Li-Fraumeni syndrome (germline variant in *TP53*) were grown in Minimum Essential Medium Eagle (Sigma; M5650) supplemented with 10% fetal calf serum, 1% glutamine, and 1% penicillin/streptomycin and cultured at 37 °C with 5% CO2. For HIPSD-seq, cells from patient LFS041 passage 62 (p.62) were harvested after trypsinizing the cells with 0.25% trypsin and resuspending them in PBS. For sci-HIPSD-seq, cells from LFS041 passage 63 (p.63) and LFS087 passage 195 (p.195) were harvested in the same way and resuspended in PBS with 1% BSA. For the mixed patient experiments for HIPSD&R-seq and DEFND-seq, cells from LFS041 passage 67 (p.67) and LFS087 passage 196 (p.196) were harvested in the same way, resuspended in PBS and 1% BSA, and mixed at 1:1 ratio. For experiments for which a direct comparison from the exact same sample source was needed (e.g., comparison between HIPSD&R-seq and DEFND), we used the same source cells. For experiments for which the direct comparison was not essential, there was a variation of one to two passages between samples.

### Nuclei extraction and nucleosome depletion for fibroblasts

For HIPSD-seq, 1 × 10^6^ cells were fixed in 1 mL of 1.5% methanol-free formaldehyde (FA; Thermo Scientific™, #28,906) in PBS, for 10 min at room temperature with gentle shaking. Fixation was neutralized by adding 200 mM glycine, followed by incubation on ice for 5 min. Cells were centrifuged (550 × g, 5 min, 4 °C) and washed with ice-cold PBS. For nuclei isolation, cells were resuspended in 1 mL of ice-cold NIB buffer (10 mM Tris–HCl pH7.4, 10 mM NaCl, 3 mM MgCl2, 0.1% igepal, 1 × protease inhibitor cocktail (#5871S, Cell Signaling Technology)) and incubated on ice for 20 min with gentle mixing. Nuclei were centrifuged (500 × g, 5 min, 4 °C) and washed once with 1X NEBuffer 2.1 (NEB, #B7202). For nucleosome depletion, nuclei were resuspended in 1X NEBuffer 2.1 supplemented with 0.3% SDS (Serva, #20,767) and incubated at 42 °C, for 15 min with shaking. SDS was quenched by adding 2% Triton-X100 (Sigma-Aldrich, #93,443), followed by incubation at 42 °C, for 15 min with shaking. Nuclei were centrifuged (500 × g, 5 min, 4 °C) and resuspended in 1X nuclei buffer (10X Genomics). Nuclei were counted on the Luna-FL™ cell counter (Logos Biosystems) and diluted to 2000–5000 nuclei/μL.

For HIPSD&R-seq, fixation with 1.5% FA, nuclei isolation with NIB buffer and nucleosome depletion with 0.3% SDS were performed exactly as in case of HIPSD-seq, with the exception that 1 U/μL of RNAse inhibitor (Takara Bio, #2313A) was added to all buffers during and after nuclei isolation (i.e., to NIB buffer and 1X NEBuffer 2.1). Following quenching of nucleosome depletion with 2% Triton-X100, nuclei samples were centrifuged (500 g, 5 min, 4 °C) and resuspended in 1X nuclei buffer (10X Genomics) containing 1 U/μL of RNAse inhibitor. Nuclei were counted using the Luna-FL™ cell counter and diluted to 2000–5000 nuclei/μL.

For sci-HIPSD-seq, cells from LFS041 (p.63) and LFS087 (p.195) were used. Fixation with 1.5% FA, nuclei isolation with NIB buffer, and nucleosome depletion with 0.3% SDS were performed exactly as in case of HIPSD-seq, with the exception that all the centrifugation steps were performed at 600 × g for 8 min at 4 °C. Following quenching of nucleosome depletion with 2% Triton-X100, nuclei samples were centrifuged (600 × g, 8 min, 4 °C) and resuspended in in ice-cold basic buffer (1 mM DTT, 2% BSA in 1 × PBS). Nuclei were counted on the Luna-FL™ cell counter. Six hundred thousand nuclei of LFS041 p.63 were used for transposition. Six thousand nuclei of LFS087 p.195 were spiked-in during transposition.

### HIPSD-seq from fibroblasts

1.1 × 10^6^ cells LFS041 p.62 were used for nuclei extraction and nucleosome depletion as described above. The nucleosome-depleted nuclei were processed using the 10X ATAC protocol, according to the Chromium Single Cell ATAC Reagent Kits User Guide v1.1 Chemistry (CG000209, 10X Genomics), but the transposase provided by 10X Genomics was replaced by highly active in-house Tn5 (83 µg/mL). Ten thousand nuclei were used for transposition and loaded on the Chromium Next GEM Chip H (PN-1000161). scDNA libraries were generated using standard reagents from the Chromium Next GEM Single Cell ATAC Library & Gel Bead Kit v1.1 (PN-1000176). Libraries were uniquely indexed with Single Index Kit N Set A (PN-1000212). Quality control and molarity calculations of the final libraries were performed with the Qubit 3.0 Fluorometer (#Q33216, Invitrogen) and the 4200 Tapestation system (Agilent Technologies). Libraries were sequenced using NovaSeq 6000 paired-end 100 SP with read 1 sequenced for 51 cycles, read 2 for 51 cycles, 8 cycles for i7, and 16 cycles for index 2. Libraries were sequenced with 1% PhiX.

### HIPSD&R-seq from fibroblasts

For the mixed patient experiment for HIPSD&R-seq from fibroblasts, 5 × 10^5^ cells each from LFS041 p.67 and LFS087 p.196 were pooled and used for nuclei extraction and nucleosome depletion as described earlier. The nucleosome depleted nuclei were processed using the 10X Multiome protocol, according to the Chromium Next GEM Single Cell Multiome ATAC + Gene Expression Reagent Kits User Guide (CG000338, 10X Genomics). Ten thousand nuclei were used for transposition and loaded on the Chromium Next GEM Chip J (PN-1000230), but the transposase provided by 10X Genomics was replaced by highly active in-house Tn5 (83 µg/mL). scDNA and scRNA libraries were generated using the standard reagents provided in the Chromium Next GEM Single Cell Multiome ATAC + Gene Expression Reagent Bundle (PN-1000285). scDNA libraries were indexed using Single Index Kit N Set A (PN-1000212), while the scRNA libraries were indexed with Dual Index Kit TT Set A (PN-1000215). Quality control and molarity calculations of the final libraries were performed with the Qubit 3.0 Fluorometer (#Q33216, Invitrogen) and the 4200 Tapestation system (Agilent Technologies). scDNA libraries were sequenced using NovaSeq 6000 (100 cycles) S1. Fifty-one cycles were used for read 1, 51 cycles for read 2, 8 cycles for i7, and 24 cycles for i5. scRNA libraries were sequenced using NovaSeq 6000 paired-end 100 SP with read 1 for 28 cycles, read 2 for 90 cycles, i7 for 10 cycles, and i5 for 10 cycles. Libraries were sequenced with 1% PhiX.

### sci-HIPSD-seq protocol with 1% spike-in (skin-derived fibroblasts)

1.66 × 10^6^ cells (LFS041 p.63) and 2.7 × 10^5^ cells (LFS087 p.195) were used for nuclei extraction and nucleosome depletion as described earlier.

#### Tn5 assembly

1.1 μL of Tn5-Read1 was mixed with 1.1 μL of each Tn5-Barcoded Read2 into 30 individual wells of a 96-well plate. For 600,000 nuclei, we used 30 Read2 barcodes (oligo list, see Additional file 2, Table S2). One microliter of barcoded annealed oligos was mixed with 1 μL of highly active in-house Tn5 into 30 wells of a new 96-well plate and Tn5 assembly was performed for 30 min at room temperature.

#### Transposition

For transposition, 2X TD was prepared by mixing equal volumes of 100% DMF and 4 × Tn5 buffer (40 mM Tris–HCl pH 7.5, 40 mM MgCl_2_). Six hundred thousand nuclei (LFS041 p.63) and 6000 nuclei (LFS087 p.195) were used for transposition in 1X TD buffer [92]. Roughly, 20,200 nuclei (18 μL of transposition mix) were added to each of the 30 wells of the 96-well plate containing 2 μL of assembled Tn5 (final dilution of Tn5 in the reaction is 1:10). The plate was incubated at 37 °C for 60 min with shaking at 500 rpm and then transferred to ice. 1 × PBS with 1% BSA (40 μL) was added to each of the wells and the samples were pooled together into Eppendorf tubes. Wells were washed with additional 40 μL of 1 × PBS with 1% BSA and transferred to the same tubes. Nuclei were collected by centrifugation (500 g, 8 min, 4 °C) and resuspended in 500 μL of 1 × PBS with 2% BSA. This nuclei suspension was filtered through a pre-wet 30 μm filter (Miltenyi Biotec, #130–041-407) to avoid clogging of the 10X chip. Filter was rinsed once with 500 μL of 1 × PBS with 0.5% BSA and collected into the sample tube. Nuclei were pelleted by centrifugation (500 g, 5 min, 4 °C), resuspended in 20 μL of 1 × nuclei buffer (10X Genomics) and counted on Luna-FL™ cell counter.

#### sci-HIPSD library preparation and sci-HIPSD-seq

This nuclei suspension was then processed using the 10X ATAC protocol, according to the Chromium Single Cell ATAC Reagent Kits User Guide v1.1 Chemistry (CG000209, 10X Genomics). A total of 180,000 nuclei were mixed with the barcoding reaction mix and loaded on the Chromium Next GEM Chip H (PN-1000161) for GEM formation. sci-HIPSD libraries were generated using standard reagents from the Chromium Next GEM Single Cell ATAC Library & Gel Bead Kit v1.1 (PN-1000176). Following the clean-up with 1.2 × AMPure XP beads, the sample was pre-amplified using Partial P5 and Partial P7 primers (oligo list, see Additional file 2, Table S2). 2.5 μL of the pre-amplified sample was used for qPCR to determine the additional number of cycles required for library amplification. The sciDNA library was then amplified using the optimal number of cycles determined from qPCR and a double size selection was performed with a 0.6 × and 1.2X ratio of AMPure XP beads (#A63882, Beckman Coulter). Quality control and molarity calculations of the final libraries were performed with the Qubit 3.0 Fluorometer (#Q33216, Invitrogen) and the 4200 Tapestation system (Agilent Technologies).

sci-HIPSD libraries were sequenced using NovaSeq 6000 paired-end 100 S1 v1.5. Three hundred picomolar was loaded and 101 cycles were used for read 1, 101 cycles for read 2, 11 cycles for i7, and 16 cycles for i5. Libraries were sequenced with 1% PhiX.

### DEFND-seq from fibroblasts

5 × 10^5^ cells consisting of a 1:1 mix of LFS041 p.67 and LFS087 p.196 were used for nucleosome depletion and library preparation with the DEFND-seq protocol [74]. For nuclei extraction and nucleosome depletion with the DEFND-seq protocol, cells were pelleted by centrifugation (500 × g, 6 min, 4 °C) and resuspended in 200 μL of DEFND buffer with RNase inhibitor (170 μL NIB, 1 × protease inhibitor cocktail (#5871S, Cell Signaling Technology), 12.5 mM lithium diiodosalicylate (#D3635, Sigma), and 1 U/μL of RNAse inhibitor (Takara Bio, #2313A)), followed by incubation on ice for 5 min. Following the incubation, 10 mL of NIB with RNase inhibitor was added and the nucleosome-depleted nuclei were pelleted by centrifugation (500 × g, 4 °C, 10 min). They were resuspended in 100 μL of 1X nuclei buffer (10X Genomics) containing 1 U/μL of RNAse inhibitor. Centrifugation step was repeated (500 × g, 4 °C, 10 min) and the pellet was resuspended in 12 μL of 1 × nuclei buffer. These nucleosome-depleted nuclei were counted using the Luna-FL™ cell counter and diluted to 1000–2000 nuclei/μL.

For library preparation, nucleosome-depleted nuclei were processed using the 10X Multiome protocol, according to the Chromium Next GEM Single Cell Multiome ATAC + Gene Expression Reagent Kits User Guide (CG000338, 10X Genomics). Five thousand nuclei were used for transposition and loaded on the Chromium Next GEM Chip J (PN-1000230), but the transposase provided by 10X Genomics was replaced by highly active in-house Tn5 (83 µg/mL). scDNA and scRNA libraries were generated using the standard reagents provided in the Chromium Next GEM Single Cell Multiome ATAC + Gene Expression Reagent Bundle (PN-1000285). scDNA libraries were indexed using Single Index Kit N Set A (PN-1000212), while the scRNA libraries were indexed with Dual Index Kit TT Set A (PN-1000215). Quality control and molarity calculations of the final libraries were performed with the Qubit 3.0 Fluorometer (#Q33216, Invitrogen) and the 4200 Tapestation system (Agilent Technologies). scDNA libraries were sequenced using NovaSeq 6000 (100 cycles) S1. Fifty-one cycles were used for read 1, 51 cycles for read 2, 8 cycles for i7, and 24 cycles for i5. scRNA libraries were sequenced using NovaSeq 6000 paired-end 100 SP with read 1 for 28 cycles, read 2 for 90 cycles, i7 for 10 cycles, and i5 for 10 cycles. Libraries were sequenced with 1% PhiX.

### Bulk whole-genome sequencing for skin-derived fibroblasts

Cell pellets from fibroblasts (LFS041 p.63 and LFS087 p.195) were prepared after trypsinization in 0.25% trypsin, cell resuspension, and centrifugation. The cell pellets were kept at − 80 °C until performing the DNA extraction using DNAeasy Blood & Tissue Kit (QIAGEN; Cat. No. ID: 69,504). Qubit Broad Range double-stranded DNA assay (Life Technologies) was used to quantify the DNA, while a Bioanalyzer (Agilent) was used to measure the quality of the DNA. Whole-genome sequencing was done using the Illumina NovaSeq 6 K paired-end 150 SP platform.

### HIPSD&R-seq in patient-derived xenograft (PDX) cells

Cryopreserved PDX cells (passage 1, primary tumor from a patient with Sonic Hedgehog medulloblastoma with germline variant in *TP53*) were thawed in a 37 °C water bath and suspended in high purity grade PBS supplemented with 10% fetal calf serum. Following suspension, the cells were centrifuged at 1000 rpm for 5 min at 4 °C and washed two times to remove any remaining DMSO. The cells were filtered with 40 µm cell strainer and brought to FACS sort in 2 mL of supplemented PBS. Directly before the FACS sort, the cells were stained with TO-PRO-3 in order to exclude any dead cells. Contaminating mouse cells were excluded based on the side forward scatter.

### Nucleosome depletion and HIPSD&R-seq library preparation (PDX cells)

A total of 420,000 FACS isolated PDX cells were used. For HIPSD&R sample preparation, fixation with 1.5% FA, nuclei isolation, and nucleosome depletion were performed exactly as in case of HIPSD&R for fibroblasts, with the exception that all centrifugation steps were performed at 800 g for 5 min at 4 °C. Following quenching of nucleosome depletion with 2% Triton-X100, nuclei were centrifuged (800 g, 5 min, 4 °C) and resuspended in 1 × nuclei buffer (10X Genomics) with 1 U/μL RNase inhibitor to a final concentration of 2000–5000 nuclei/μL. Ten thousand nuclei were used for transposition and HIPSD&R library preparation following the exact 10X Multiome protocol mentioned in case of HIPSD&R from fibroblasts.

scDNA libraries were sequenced using NovaSeq 6000 (138 cycles) SP, with 51 cycles for read 1, 51 cycles for read 2, 8 cycles for index 1, and 24 cycles for index 2. scRNA libraries were sequenced using NovaSeq 6000 (138 cycles) SP, with 28 cycles for read 1, 90 cycles for read 2, 10 cycles for i7, and 10 cycles for i5. Libraries were sequenced with 1% PhiX.

### 10X CNV single-cell DNA sequencing library preparation (PDX cells)

The single-cell suspensions from PDX cells were processed using the Chromium Single-Cell CNV Kit (10 × Genomics) [87] according to the manufacturer’s protocol. In brief, using cell bead polymer, single cells were partitioned in a hydrogel matrix on Chromium Chip C. Once the cell beads were encapsulated and incubated, they were subjected to enzymatic and chemical treatment. This lysed the encapsulated cells and denatured the gDNA in the cell bead, to make it accessible for further amplification and barcoding. A second encapsulation was performed to achieve single cell resolution by co-encapsulating a single cell bead and a single barcoded gel bead to generate GEMs. Immediately after GEM generation, the gel bead and cell bead were dissolved. Oligonucleotides containing standard Illumina adaptors and 10 × barcoded fragments were then amplified with 14 PCR cycles during two-step isothermal incubation. After incubation, the GEMs were broken and pooled 10 × barcoded fragments were recovered. Single-cell libraries were sequenced on the Illumina NextSeq platform (150 bp, paired-end). For more details see Smirnov et al. [88].

### 10 × Chromium single-cell ATAC

Cells (1:1 mix of fibroblasts from LFS patient 41 p.67 and patient 87 p.196) were treated with 100 µL chilled lysis buffer (10 mM Tris pH7.4, 10 mM NaCl, 3 mM MgCl2, 1% BSA, 0.1% Tween-20, 0.1% Nonident P40, 0.01% digitonin) for 5 min on ice. One milliliter chilled wash buffer (10 mM Tris pH7.4, 10 mM NaCl, 3 mM MgCl2, 1% BSA, 0.1% Tween-20) was added and nuclei were centrifuged for 5 min at 500 rcf. Nuclei were resuspended in a diluted nuclei buffer as provided in the ATAC kit, counted and further processed using the standard 10 × ATAC protocol according to the user guides for Chromium Next GEM Single Cell ATAC v2.

### Computational analyses

The reference genome GRCh38 was used for all analyses. For all single cell experiments, the reference provided by 10 × Genomics (Cell Ranger Arc, GRCh38, version 2020-A) was used.

#### Analysis of bulk whole-genome sequencing data

Reads were aligned with BWA-MEM (version 0.7.15) [93]. Duplicates were marked with Picard (version 2.25.1) [94] and later removed with SAMtools [95] together with low-quality reads. High-quality read counts are extracted with hmmcopy_utils [96] at different bin sizes (10 kb, 50 kb, 100 kb, 500 kb, or 1 MB).

#### HIPSD-seq raw data preprocessing

The initial preprocessing of HIPSD-seq and sci-HIPSD-seq samples is performed using the 10 × Cellranger ATAC pipeline (version 2.1.0) [97], while 10 × Cellranger Arc (version 2.0.2) is used for processing of the DNA component of HIPSD&R-seq and DEFND-seq. To avoid defining cells based on peak calling results, we define cells based on the number of fragments per cell. Briefly, based on a spline curve fitted to a log-transformed barcode rank plot (log(fragments) vs. log(rank)), we take all the barcodes with fragment count above the value of the highest gradient of the curve (Cell Ranger v7.2.0). We provide our custom implementation of cell calling based on the barcode rank plot gradient in https://github.com/OtonicarJan/HIPSDR-seq [98]. In the sciHIPSD-seq experiment with sample LFS041_LFS087_spikein and HIPSD&R-seq experiment with PDX, a cutoff of 10,000 fragments was used to define cells.

When the cell barcodes are retrieved, we extract cell-specific BAM files from Cellranger produced BAM files using subset-bam (version 1.1.0) [99] tool from 10 × Genomics. Individual BAM files are preprocessed by removing low-quality reads and duplicates (using SAMtools [95]) and then counts are extracted using hmmcopy_utils at different bin sizes (10 kb, 50 kb, 100 kb, 500 kb, or 1 MB) [100]. Finally, all the nuclei with less than 90% of non-empty genomic bins at 1 MB bin size are filtered out (the total number of 1 MB genomic bins is 3102).

#### Metacelling

To perform high confidence copy number calling on 100 kb genomic windows, we aggregate cells into metacells. The aggregation is performed in the following steps:

1. Cell counts are aggregated into 1 MB windows to increase signal-to-noise ratio and reduce sparsity.
2. Principal components are computed to reduce dimensionality, while preserving 95% of variance.
3. Cells are preclustered using Leiden clustering (based on the principal components) such that the maximal number of reads per 1 MB does not exceed 1200 (can be selected by user). This step creates initial metacells that should be fine-grain enough to avoid clamping together genetically distinct cells.
4. Premetacells are separated into three categories: those with insufficient coverage (selected by user—200 reads per 1 MB in our case that matches 0.02 × coverage), those with sufficient coverage, those with high coverage (over 1200 reads per 1 MB).
5. A greedy algorithm ranks every cell pair by genetic distance and makes a full pass merging premetacells within fixed distance (selected by a user) if both cells have insufficient coverage, or one cell has insufficient coverage and one sufficient. Premetacells with high coverage are not merged with any other cells as we want to avoid creating cells with a coverage that is way higher than others.
6. Steps 4 and 5 are repeated until there are no more possible merges.

Selecting appropriate merging distance for step 5 is essential. The value is chosen such that the trade-off between within metacell variation (high with large distances) and unmerged cells (high with small distances) is optimal. In our case, we computed 10 quantiles of all distances between premetacells and picked as the final merging distance the one that gives the best within metacell variation vs. number of unmerged cells trade-off.

Additional file 1: Fig. S9 shows the metacelling steps and results using as an example HIPSD-seq sample from LFS041 p.62. The code is available at https://github.com/OtonicarJan/HIPSDR-seq [98].

#### Copy number calling for scDNA-seq data

For scDNA-seq data (HIPSD-seq, sciHIPSD-seq, and DEFND-seq) and bulk WGS data, we called copy number profiles using HMMcopy tool [101]. To enable confident CNV calling for bin sizes below 1 MB while preserving as many cells as possible, we applied metacelling to scDNA-seq to increase the coverage per cell (see Additional file 1: Fig. S3 for pre-metacelling coverage and post-metacelling coverage, as well as cell per metacell assignment). For bin sizes 1 MB and 500 kb, HMMcopy parameter e (the suggested probability to stay in a segment) was set to 0.995. For bin sizes 100 kb, 50 kb, and 10 kb, HMMcopy parameter e was set to 0.999999999999999 and strength parameter to 1e30. We noticed the tendency of HMMcopy to overcall homozygous deletions for all datasets regardless of coverage and therefore we do not distinguish homozygous and heterozygous deletions in our results.

For all scDNA-seq and scATAC-seq datasets, k-means was used for unsupervised clustering of the nuclei based on the CNV estimates with k = 2. The exception is sciHIPSD-seq for which Leiden clustering was used with the resolution set to 0.15.

We used Pearson’s correlation coefficient to calculate the correlation of CNV estimates from single cells or metacells to CNV estimates from bulk WGS data. In all method comparisons, the maximum number of overlapping genomic bins between the two assays was used. The exception is Fig. 2H, where for each of the assays the maximum number of overlapping genomic bins with the bulk WGS data was used.

In all the heatmaps of HIPSD&R-seq or DEFND-seq datasets, only the barcodes that were kept after filtering based on non-empty bins and are also found in the RNA component are shown.

#### scRNA-seq data analysis

scRNA data was processed using the Cellranger pipeline (version 7.1.0) [67]. For the analysis of fibroblast data, we used Numbat (version 1.4.0) [49] with default parameters using publicly available data from BJ fibroblasts (SRR23292070) [102] as a reference to estimate copy numbers from scRNA-seq data. For analysis of PDX data, Numbat [49] with default parameters and reference was used. In Fig. 2, we mapped normalized gene expressions from Numbat to fixed genomic bins, adjusting the bin size. In cases where there was more than one gene per genomic bin, the mean of normalized gene expressions was used. Nuclei were clustered with k-means and k = 2 based on the normalized gene expression mapped to genomic bins, except for Fig. 2H and Additional file 1: Fig. S6, where k-means was used on the normalized gene expressions. Since Numbat performs allele-specific copy number calling, but we have a mix of fibroblasts from two different patients, we could not use the main output from Numbat—allele-specific CNVs—for downstream analyses. Downstream analysis was performed with Scanpy (version 1.10.0) [103] following single-cell best practices [104]. Briefly, outlier cells based on total number of counts per cell, number of genes per cell, and percentage of counts mapping to mitochondrial genes were removed. Next, ambient RNA correction was performed with SoupX (version 1.6.2) [105], followed by doublet detection with scDblFinder (version 1.14.0) [106]. After doublet removal, counts were normalized and log-transformed. Next, PCA was performed on the most variably expressed genes and first 15 PCs were used to construct a KNN graph. Clusters from scDNA-seq were mapped to the matching nuclei on scRNA level. Finally, UMAP was used to visualize nuclei in two dimensions.

To identify differentially expressed genes between the clusters, rank_genes_groups function from Scanpy with Wilcoxon rank sum method was used. Results were filtered further with function filter_rank_genes_groups selecting only differentially expressed genes with fold change higher than 1, expressed in more than 25% of the cells in the test cluster and in less than 50% of cells in the compared cluster.

#### Resolution analysis of CNV estimates

As genome-wide correlations only provide an overall match between single cells/metacells and bulk, we designed an analysis that would enable us to compare CNVs in smaller genomic regions. The resolution analysis of CNV estimates was performed on the cluster 0 of metacells and single cells of the DNA component of HIPSD&R-seq and on the cluster 0 of single cells of the RNA component of HIPSD&R-seq. All the mentioned CNV estimates were compared to the WGS-derived CNV profile of fibroblasts from the patient LFS087 (p.195). First, CNVs from WGS and the DNA component of HIPSD&R-seq were encoded as either a loss, neutral, or a gain. Smoothed expressions from Numbat of the RNA component were first mapped to fixed genomic bins of various sizes (100 kb, 500 kb, or 1 Mb) and then classified as either a loss, diploid, or a gain based on the fraction of losses and gains in the bulk CNV profile of the patient LFS087 (p.195). For example, if the bulk profile had 25% of genomic bins with losses and 25% with gains, then in each cell, smoothed expressions up to the first quartile would be classified as a loss, from the first quartile to the third quartile as diploid and from the third quartile on as a gain. Only genomic bins that overlap between different assays were used.

Next, a non-overlapping sliding window of various sizes (5 bins, 10 bins, 50 bins, and 100 bins) was applied across the genome to CNVs computed at different bin sizes (100 kb, 500 kb, and 1 Mb), where at each window position, a macro F1 score was calculated between each single cell/metacell and bulk profile. At each window position, an average macro F1 score across all the cells/metacells in the assay and cluster was reported. Window positions with only diploid states were skipped. For this analysis, we also used two computationally designed controls: completely diploid profile (all bins with diploid state) and random control, for which we artificially generated 500 profiles with the same fractions of losses and gains as the bulk WGS profile, but randomly shuffled.

#### scATAC-seq data analysis

Reads were processed with the 10X Cellranger ATAC pipeline (version 2.1.0). We used Muon (version 0.1.6) [107] for the downstream analysis as well as filtering out nuclei with fragment count under 20,000 and TSS enrichment score under 3. CNVs were inferred for high-quality nuclei (*N* = 12,496) with epiAneufinder (version 1.0.3) using the window size of 1 MB [89]. Pearson’s correlation coefficient was used to calculate the correlation of CNVs per nuclei inferred with epiAneufinder, to bulk CNVs.

## Supplementary Information

Additional file 1: Fig. S1. Comparison between DEFND-seq and HIPSD&R-seq (DNA components, for the same sample, 1:1 mix of fibroblasts from two patients). (A) Throughput and TSS enrichment score. (B, C) Barcode rank plots showing all barcodes detected in comparative HIPSD&R-seq and DEFND-seq experiment for the DNA component. The blue lines show detected nuclei and the gray lines show the background. The input material was the same for both methods and the libraries were sequenced with the same sequencing depth. The highest gradient in the barcode rank plot is used as a threshold to differentiate nuclei from the background. (D, E) Correlations of CNV estimates from DEFND-seq (DNA component, panel D) and from HIPSD&R-seq (panel E) to bulk WGS, respectively. The correlation with bulk WGS is higher for HIPSD&R-seq as compared to DEFND. Unsupervised clustering was performed on CNVs to identify two clusters, corresponding to two patients. (F–I) Heatmaps show CNVs for DEFND-seq and for HIPSD&R-seq for the same sample. Each row represents a single nucleus in G and I and one metacell in F and H. HMMcopy was used (1 MB bin size for single cells and 100 kb for metacells). Blue, pink, brown, and orange labels are based on the unsupervised clustering shown in D and E. HIPSD&R-seq provides more accurate copy-number inference as compared to DEFND-seq. Fig. S2. Comparison of performance parameters between HIPSD&R-seq and DEFND-seq RNA components, for the same sample (1:1 mix of fibroblasts from two patients). (A) Basic performance parameters. (B–E) Barcode rank plots showing all barcodes detected in our own comparative HIPSD&R-seq and DEFND-seq experiment as well as in public DEFND data (D) BJ fibroblasts (SRR23292070) [102] and (E) glioblastoma (SRR23292060) [111]. The blue lines show detected nuclei and the gray lines show the background. For the comparative HIPSD&R-seq and DEFND-seq experiment, the input material was the same for both methods and the libraries were sequenced with the same depth. Right, contamination fraction computed with SoupX. The contamination fraction is shown on the x-axis, with 0 meaning no contamination and 1 meaning that 100% of UMIs in a droplet are soup. Fig. S3. Assay properties and quality metrics on nuclei and metacells (HIPSD&R-seq (PDX), HIPSD&R-seq (LFS041_LFS087_1:1), HIPSD-seq (LFS041_p62), sciHIPSD-seq (LFS041_LFS087_spikein), DEFND-seq (LFS041_LFS087_1:1), and Chromium Single Cell CNV (PDX) sample). (A) Read coverage of all recovered nuclei HIPSD&R-seq/HIPSD-seq/sciHIPSD-seq/DEFND-seq, and for data from the 10x CNV kit. (B) Read coverage of the nuclei before and after filtering based on non-empty bins for HIPSD&R-seq/sciHIPSD-seq/DEFND-seq (after filtering 4490/17,319/1268 nuclei are kept). (C) Read coverage of metacells for HIPSD&R-seq/HIPSD-seq/DEFND-seq. (D) Cumulative read counts of all recovered nuclei for HIPSD&R-seq/HIPSD-seq/sciHIPSD-seq/DEFND-seq and 10x CNV kit. (E) Cumulative read counts of the nuclei before and after filtering based on non-empty bins for HIPSD&R-seq/sciHIPSD-seq/DEFND-seq. (F) Cumulative counts distribution with metacelling for HIPSD&R-seq/HIPSD-seq/DEFND-seq. (G) Duplication rate in single cells. (H) Number of cells per metacell. (I) Lorenz curves to assess the uniformity of the coverage for nuclei before and after filtering based on non-empty bins for HIPSD&R-seq/sciHIPSD-seq/DEFND-seq. (J) Sequencing saturation for the HIPSD&R-seq RNA component versus conventional 10x 3′ scRNA-seq on the original cells of the PDX sample. The total number of nuclei and metacells is as follows: HIPSD&R-seq (PDX)—2658 nuclei/101 metacells, HIPSD&R-seq (LFS041_LFS087_1:1)—7847 nuclei/299 metacells, HIPSD-seq (LFS041_p62)—5084 nuclei/492 metacells, sciHIPSD-seq (LFS041_LFS087_spikein)—50,359 nuclei, DEFND-seq (LFS041_LFS087_1:1)—1282 nuclei/636 metacells, and Chromium Single Cell CNV (PDX) sample—244 nuclei. Fig. S4. HIPSD&R-seq shows close correspondence to the reference bulk data (patient-derived xenograft from a medulloblastoma). From top to bottom: CNV estimates derived from bulk WGS data (top), CNV estimates derived from HIPSD&R-seq in 101 metacells (middle), CNV estimates derived from the HIPSD&R-seq RNA component (bottom, numbat window-smoothed expression). DNA-based CNV estimates obtained using HMMcopy with 100 kb bin size; RNA-based CNV estimates derived using Numbat. Right: Zoom-in view on chromosome 5, depicting a representative region where CNV estimates derived from RNA failed to capture a localized amplification event (marked in red). Fig. S5. Comparison between ATAC-seq and HIPSD&R-seq (DNA component, for the same sample, 1:1 mix of fibroblasts from two patients). (A) Basic performance parameters and TSS enrichment scores. Note: Whereas in ATAC the median TSS enrichment score is around 3.4, as expected for ATAC data, the TSS scores for HIPSD&R-seq and for sciHIPSD are below 2 and below 1, respectively, showing that no significant ATAC-dependent bias remains. (B) Fragment length distribution for ATAC-seq has a clear periodicity, as expected. However, this periodicity is not observed in HIPSD&R or sciHIPSD-seq data, showing no ATAC-dependent bias. (C, D) Heatmaps show copy-number variation for ATAC-seq (C) and HIPSD&R-seq. Each row shows one single nucleus. For ATAC-seq, epiAneufinder was used and for HIPSD&R-seq HMMCopy was used (1 MB bin size for both). Blue and pink labels shown in the right panels (E and F) are based on unsupervised clustering of CNVs from nuclei. HIPSD&R-seq provides more accurate copy-number inference as compared to ATAC-seq. (E, F) Correlation of CNVs from HIPSD&R-seq (DNA component) and from ATAC-seq to bulk WGS, respectively. The correlation with bulk WGS is higher for HIPSD&R-seq as compared to copy-number inference from ATAC-seq. Fig. S6. Resolution analysis of CNV estimates from DNA and RNA components of HIPSD&R-seq. (A) CNV estimates from metacells of DNA component of cluster 0 and cells of RNA component of cluster 0, both from Fig. 2, were correlated to CNV estimates of bulk WGS data of the patient LFS087. Shown are Pearson’s correlation coefficients for pairs of alternative methods with respect to bulk WGS. For each bin size, only genomic bins that overlap between both components of HIPSD&R-seq were used to calculate correlations. (B) Schematic overview of resolution analysis shown in (C), where a non-overlapping sliding window with various sizes is used to calculate an average F1 score between CNV estimates from single cells/metacells and CNV estimates for a reference bulk profile. An average F1 score across all cells/metacells is reported for a single window position. Windows that are fully diploid in the bulk are skipped as the focus of this analysis is on altered regions. (C) Average F1 score between CNV estimates of bulk WGS data of the patient LFS087 and CNV estimates from metacells, single cells (DNA and RNA) of cluster 0, random control cells, or fully diploid cells (see[e1]  “Methods”) for bin sizes 100 kb, 500 kb, and 1 Mb. X axes represent the size of a sliding window. Fig. S7. Analysis of differentially expressed genes between clusters in HIPSD&R-seq. UMAPs of scDNA-seq based on CNVs (A) and scRNA-seq based on gene expressions (B) (DNA and RNA components from HIPSD&R-seq). Points are colored based on unsupervised clustering of CNVs from scDNA-seq from fibroblasts from patient 41 and patient 87 mixed at a 1:1 ratio. Clusters remain well separated, showing that cells from patient 41 (cluster 1) are relatively homogenous at DNA and RNA level as compared to cells from patient 87, which comprise three major groups of cells retrieved at DNA level that also make three main groups of cells at RNA level. (C) Dot plot showing top 10 differentially expressed genes between cells from cluster 0 (correlated to male patient 87) and cluster 1 (correlated to female patient 41) from HIPSD&R-seq data. Sex specific genes (e.g., *XIST*) as well as genes located in chromosome regions for which the copy number differs between both patients are among the top differentially expressed genes and are circled. Fig. S8. sciHIPSD-seq identifies a rare clone. UMAP with the two Leiden clusters from Fig. 3. The mixed suspension with nuclei from both patients was analyzed using sciHIPSD-seq and the 1% clone can be robustly identified by Leiden clustering of CNVs. Fig. S9. Metacelling workflow. (A) Pre metacelling results: copy number called on 100 kb resolution for bulk data (top) and single cell (bottom); counts UMAP for 1 MB window. (B) Cells are preclustered to show candidate metacells for which some of the clusters might not have sufficient coverage. (C) Pre-metacells are greedily merged within a fixed distance until no metacell has a coverage lower than a selected threshold. (D) Copy number called on metacells (bottom) and corresponding bulk with fewer mismatches (top); comparison between bulk, initial cells and metacells shows that metacells produce closer match to bulk. [e1]CE: Please cross-link this section citation.

Additional file 2: Table S1. Samples characteristics. List of samples used in this study and key QC metrics. This includes the tissue or sample type description; passage (applicable only to fibroblast samples); assay used; number of cells obtained based on the number of fragments for DNA data or based on cellranger definition for RNA data; number of cells in the DNA component after filtering; total number of read pairs; median reads per cell, genes per cell, duplication, and sequencing saturation. Table S2. List of oligos used in the HIPSD-seq and sci-HIPSD-seq assays.

Additional file 3. Review history.

## Data Availability

All raw sequencing data for this study is available at the European Genome-Phenome Archive (EGA) under accession number EGAS50000000691 [108]. 10 × Genomics CNV and RNA data is obtained from Smirnov et al. [88] and available from EGA under accession number EGAS00001005410 [109]. The code to reproduce presented results is available at GitHub repository https://github.com/OtonicarJan/HIPSDR-seq [98]. The source code used in the manuscript was also deposited in Zenodo (10.5281/zenodo.14011821) [110]. The source code on GitHub and Zenodo is released under an MIT license.
