# Supplementary material for: HIPSD&R-seq enables scalable genomic copy number and transcriptome profiling
Source: Genome Biol. 2024 Dec 18;25:316. doi: 10.1186/s13059-024-03450-0 (PMC11657747; doi:10.1186/s13059-024-03450-0)
Supplement: Supplementary file 1 — Additional file 1: Fig. S1. Comparison between DEFND-seq and HIPSD&R-seq (DNA components, for the same sample, 1:1 mix of fibroblasts from two patients). (A) Throughput and TSS enrichment score. (B, C) Barcode rank plots showing all barcodes detected in comparative HIPSD&R-seq and DEFND-seq experiment for the DNA component. The blue lines show detected nuclei and the gray lines show the background. The input material was the same for both methods and the libraries were sequenced with the same sequencing depth. The highest gradient in the barcode rank plot is used as a threshold to differentiate nuclei from the background. (D, E) Correlations of CNV estimates from DEFND-seq (DNA component, panel D) and from HIPSD&R-seq (panel E) to bulk WGS, respectively. The correlation with bulk WGS is higher for HIPSD&R-seq as compared to DEFND. Unsupervised clustering was performed on CNVs to identify two clusters, corresponding to two patients. (F–I) Heatmaps show CNVs for DEFND-seq and for HIPSD&R-seq for the same sample. Each row represents a single nucleus in G and I and one metacell in F and H. HMMcopy was used (1 MB bin size for single cells and 100 kb for metacells). Blue, pink, brown, and orange labels are based on the unsupervised clustering shown in D and E. HIPSD&R-seq provides more accurate copy-number inference as compared to DEFND-seq. Fig. S2. Comparison of performance parameters between HIPSD&R-seq and DEFND-seq RNA components, for the same sample (1:1 mix of fibroblasts from two patients). (A) Basic performance parameters. (B–E) Barcode rank plots showing all barcodes detected in our own comparative HIPSD&R-seq and DEFND-seq experiment as well as in public DEFND data (D) BJ fibroblasts (SRR23292070) [102] and (E) glioblastoma (SRR23292060) [111]. The blue lines show detected nuclei and the gray lines show the background. For the comparative HIPSD&R-seq and DEFND-seq experiment, the input material was the same for both methods and the libraries were sequenced with [file 13059_2024_3450_MOESM1_ESM.pdf]

A

Method	Number of nuclei	Median of total reads per nucleus	Median of non-empty bins per nucleus (1MB)	Median TSS score enrichment on putative cells
DEFND-seq	1,282	623,644	2,920	1.10
HIPSD&R-seq	7,847	49,233	2,826	1.88

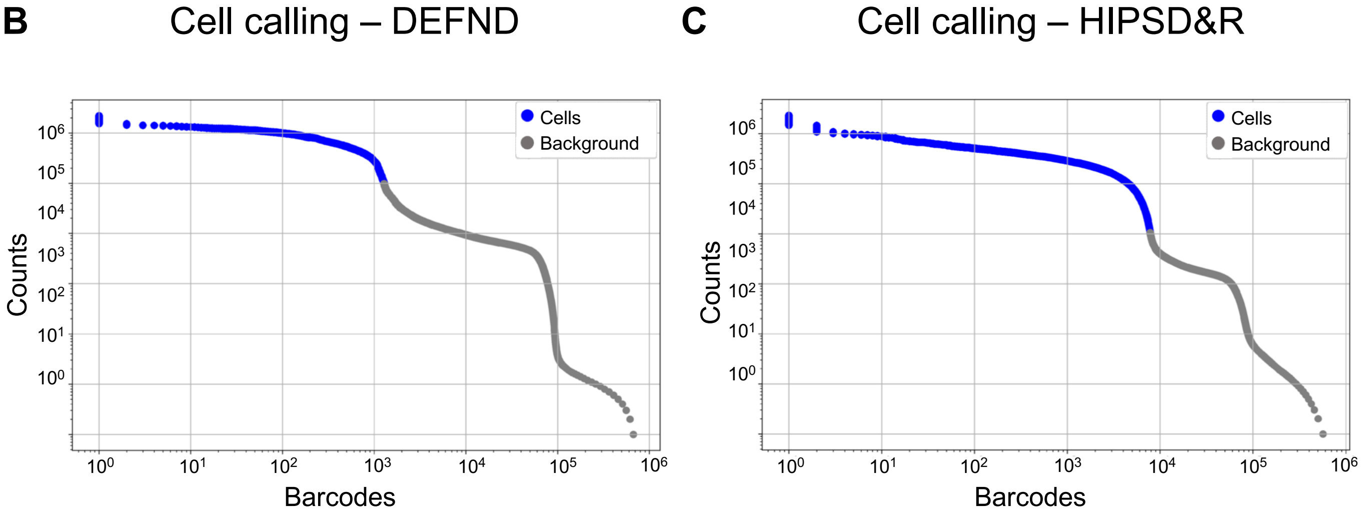

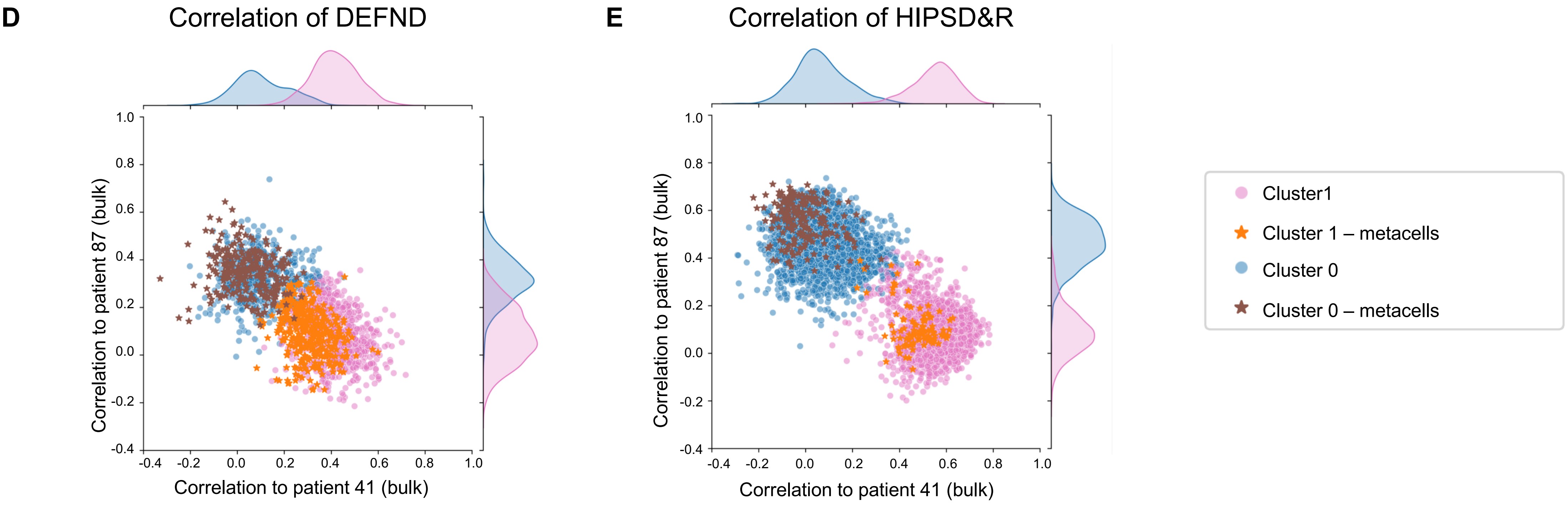

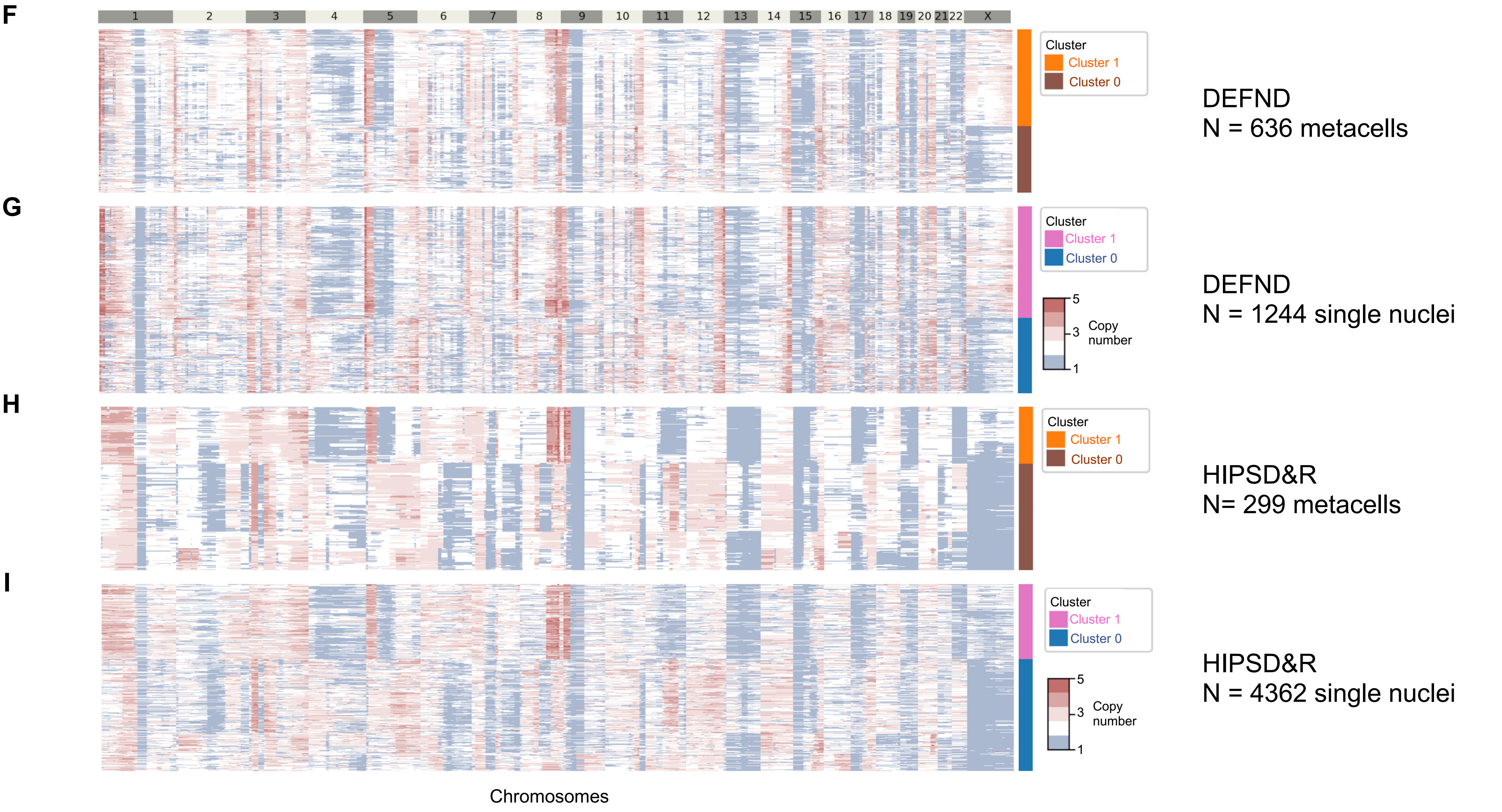

Fig. S1 | Comparison between DEFND-seq and HIPSD&R-seq (DNA components, for the same sample, 1:1 mix of fibroblasts from two patients). (A) Throughput and TSS enrichment score. **(B, C)** Barcode rank plots showing all barcodes detected in comparative HIPSD&R-seq and DEFND-seq experiment for the DNA component. The blue lines show detected nuclei and the grey lines show the background. The input material was the same for both methods and the libraries were sequenced with the same sequencing depth. The highest gradient in the barcode rank plot is used as a threshold to differentiate nuclei from the background. **(D, E)** Correlations of CNV estimates from DEFND-seq (DNA component, panel D) and from HIPSD&R-seq (panel E) to bulk WGS, respectively. The correlation with bulk WGS is higher for HIPSD&R-seq as compared to DEFND. Unsupervised clustering was performed on CNVs to identify two clusters, corresponding to two patients. **(F-I)** Heatmaps show CNVs for DEFND-seq and for HIPSD&R-seq for the same sample. Each row represents a single nucleus in G and I and one metacell in F and H. HMMcopy was used (1MB bin size for single cells and 100kb for metacells). Blue, pink, brown and orange labels are based on the unsupervised clustering shown in **D** and **E.** HIPSD&R-seq provides more accurate copy-number inference as compared to DEFND-seq.

A

Sample Name	Method	Estimated Number of Nuclei (total)	Mean Reads per Nucleus	Median counts per nucleus	Median Genes per Nucleus (GRCh38)	Median % of mitochondrial counts per nucleus	Median % of ribosomal counts per nucleus	Valid Barcodes
HIPSD&R_GEX	HIPSD&R-seq	8,764	58,133	3,890.5	1,990	1.99	14.3	93.1%
DEFND_GEX	DEFND-seq	2,457	153,895	7,618	3,637	0.75	4.2	93.2%

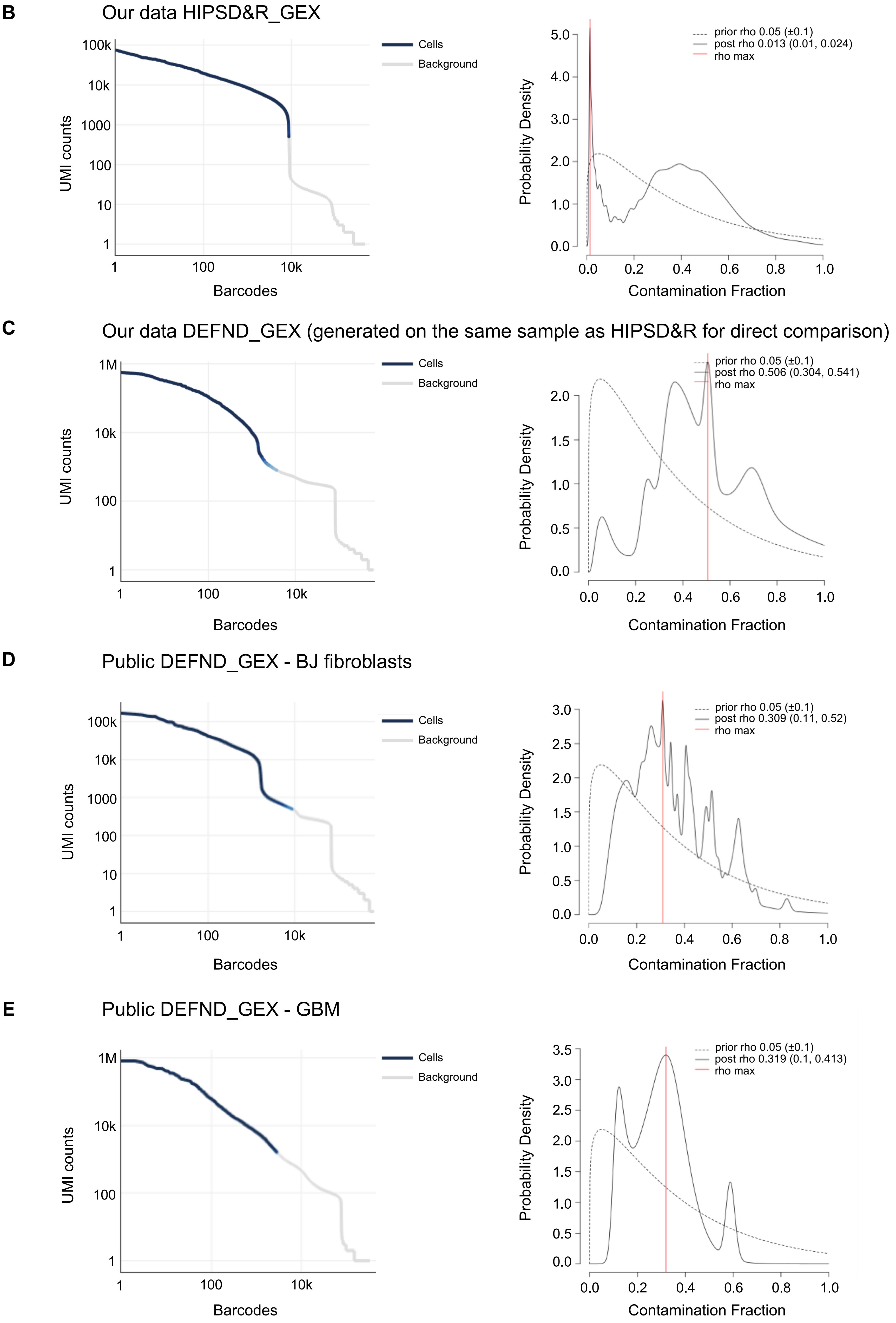

Fig. S2 | Comparison of performance parameters between HIPSD&R-seq and DEFND-seq RNA components, for the same sample (1:1 mix of fibroblasts from two patients). (A) Basic performance parameters. **(B-E)** Barcode rank plots showing all barcodes detected in our own comparative HIPSD&R-seq and DEFND-seq experiment as well as in public DEFND data (D) BJ fibroblasts (SRR23292070) and (E) glioblastoma (SRR23292060). The blue lines show detected nuclei and the grey lines show the background. For the comparative HIPSD&R-seq and DEFND-seq experiment, the input material was the same for both methods and the libraries were sequenced with the same depth. Right, contamination fraction computed with SoupX. The contamination fraction is shown on the x-axis, with 0 meaning no contamination and 1 meaning that 100% of UMIs in a droplet are soup.

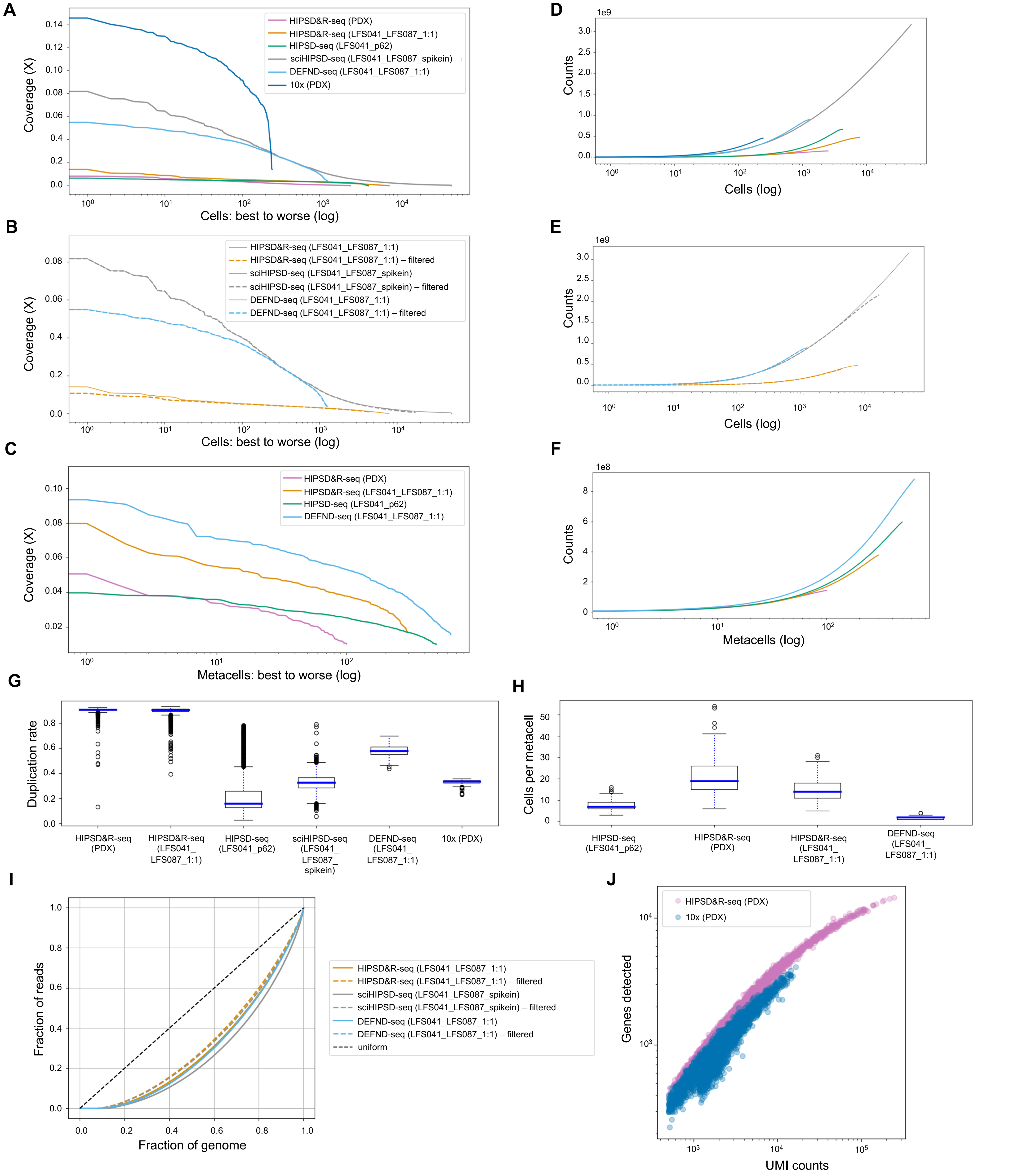

Fig. S3 | Assay properties and quality metrics on nuclei and metacells (HIPSD&R-seq (PDX), HIPSD&R-seq (LFS041_LFS087_1:1), HIPSD-seq (LFS041_p62), sciHIPSD-seq (LFS041_LFS087_spikein), DEFND-seq (LFS041_LFS087_1:1) and Chromium Single Cell CNV (PDX) sample). (**A**) Read coverage of all recovered nuclei HIPSD&R-seq/HIPSD-seq/sciHIPSD-seq/DEFND-seq, and for data from the 10x CNV kit. (**B**) Read coverage of the nuclei before and after filtering based on non-empty bins for HIPSD&R-seq/sciHIPSD-seq/DEFND-seq (after filtering 4,490/17,319/1,268 nuclei are kept). (**C**) Read coverage of metacells for HIPSD&R-seq/HIPSD-seq/DEFND-seq. (**D**) Cumulative read counts of all recovered nuclei for HIPSD&R-seq/HIPSD-seq/sciHIPSD-seq/DEFND-seq and 10x CNV kit. (**E**) Cumulative read counts of the nuclei before and after filtering based on non-empty bins for HIPSD&R-seq/sciHIPSD-seq/DEFND-seq. (**F**) Cumulative counts distribution with metacelling for HIPSD&R-seq/HIPSD-seq/DEFND-seq. (**G**) Duplication rate in single cells. (**H**) Number of cells per metacell. (**I**) Lorenz curves to assess the uniformity of the coverage for nuclei before and after filtering based on non-empty bins for HIPSD&R-seq/sciHIPSD-seq/DEFND-seq. (**J**) Sequencing saturation for the HIPSD&R-seq RNA component versus conventional 10x 3' scRNA-seq on the original cells of the PDX sample. The total number of nuclei and metacells is: HIPSD&R-seq (PDX) - 2,658 nuclei/101 metacells, HIPSD&R-seq (LFS041_LFS087_1:1) - 7,847 nuclei/299 metacells, HIPSD-seq (LFS041_p62) - 5,084 nuclei/492 metacells, sciHIPSD-seq (LFS041_LFS087_spikein) - 50,359 nuclei, DEFND-seq (LFS041_LFS087_1:1) - 1,282 nuclei/636 metacells and Chromium Single Cell CNV (PDX) sample - 244 nuclei.

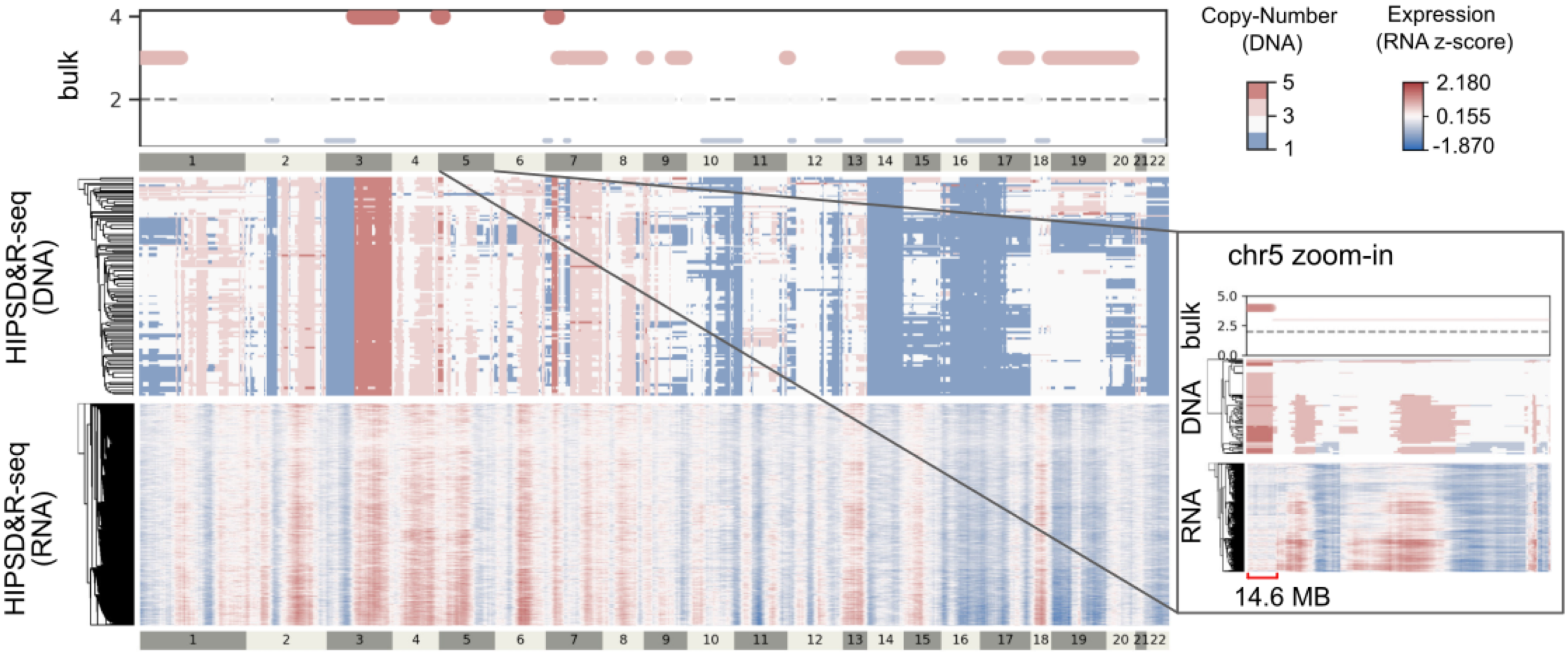

Fig. S4 | HIPSD&R-seq shows close correspondence to the reference bulk data (patient-derived xenograft from a medulloblastoma). From top to bottom: CNV estimates derived from bulk WGS data (top), CNV estimates derived from HIPSD&R-seq in 101 metacells (middle), CNV estimates derived from the HIPSD&R-seq RNA component (bottom, Numbat window-smoothed expression). DNA-based CNV estimates obtained using HMMcopy with 100kb bin size; RNA-based CNV estimates derived using Numbat. Right: Zoom-in view on chromosome 5, depicting a representative region where CNV estimates derived from RNA failed to capture a localised amplification event (marked in red).

A

Method	Number of nuclei	Median of total reads per nucleus	Median of non-empty bins per nucleus (1MB)	Median TSS score enrichment on putative cells
HIPSD&R-seq	7,847	49,233	2,826	1.88
ATAC-seq	20,000 *	41,262 **	/	3.41
sciHIPSD-seq	50,359	28,749	2,715	0.95

* The number of nuclei for ATAC is larger as compared to HIPSD&R because we loaded more nuclei for ATAC.

** For ATAC, we provide the number of high-quality fragments per cell, not reads per cell.

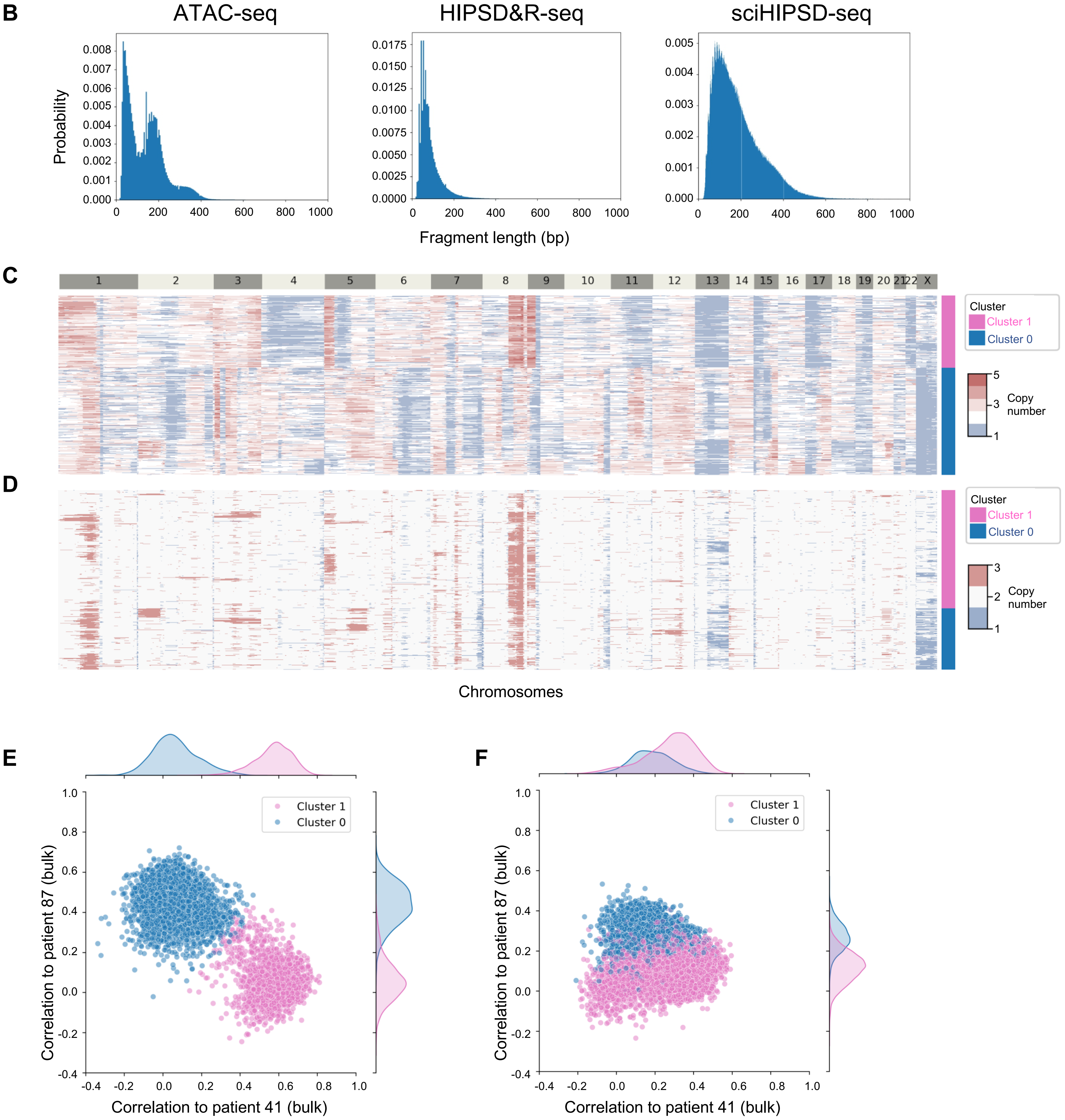

Fig. S5 | Comparison between ATAC-seq and HIPSD&R-seq (DNA component, for the same sample, 1:1 mix of fibroblasts from two patients). (A) Basic performance parameters and TSS enrichment scores. Note: Whereas in ATAC the median TSS enrichment score is around 3.4, as expected for ATAC data, the TSS scores for HIPSD&R-seq and for sciHIPSD are below 2 and below 1, respectively, showing that no significant ATAC-dependent bias remains. **(B)** Fragment length distribution for ATAC-seq has a clear periodicity, as expected. However, this periodicity is not observed in HIPSD&R or sciHIPSD-seq data, showing no ATAC-dependent bias. **(C, D)** Heatmaps show copy-number variation for HIPSD&R-seq **(C)** and ATAC-seq **(D)**. Each row shows one single nucleus. For ATAC-seq, epiAneufinder was used and for HIPSD&R-seq HMMCopy was used (1MB bin size for both). Blue and pink labels shown in the right panels **(E** and **F)** are based on unsupervised clustering of CNVs from nuclei. HIPSD&R-seq provides more accurate copy-number inference as compared to ATAC-seq. **(E, F)** Correlation of CNVs from HIPSD&R-seq **(E)** (DNA component) and from ATAC-seq **(F)** to bulk WGS, respectively. The correlation with bulk WGS is higher for HIPSD&R-seq as compared to copy-number inference from ATAC-seq.

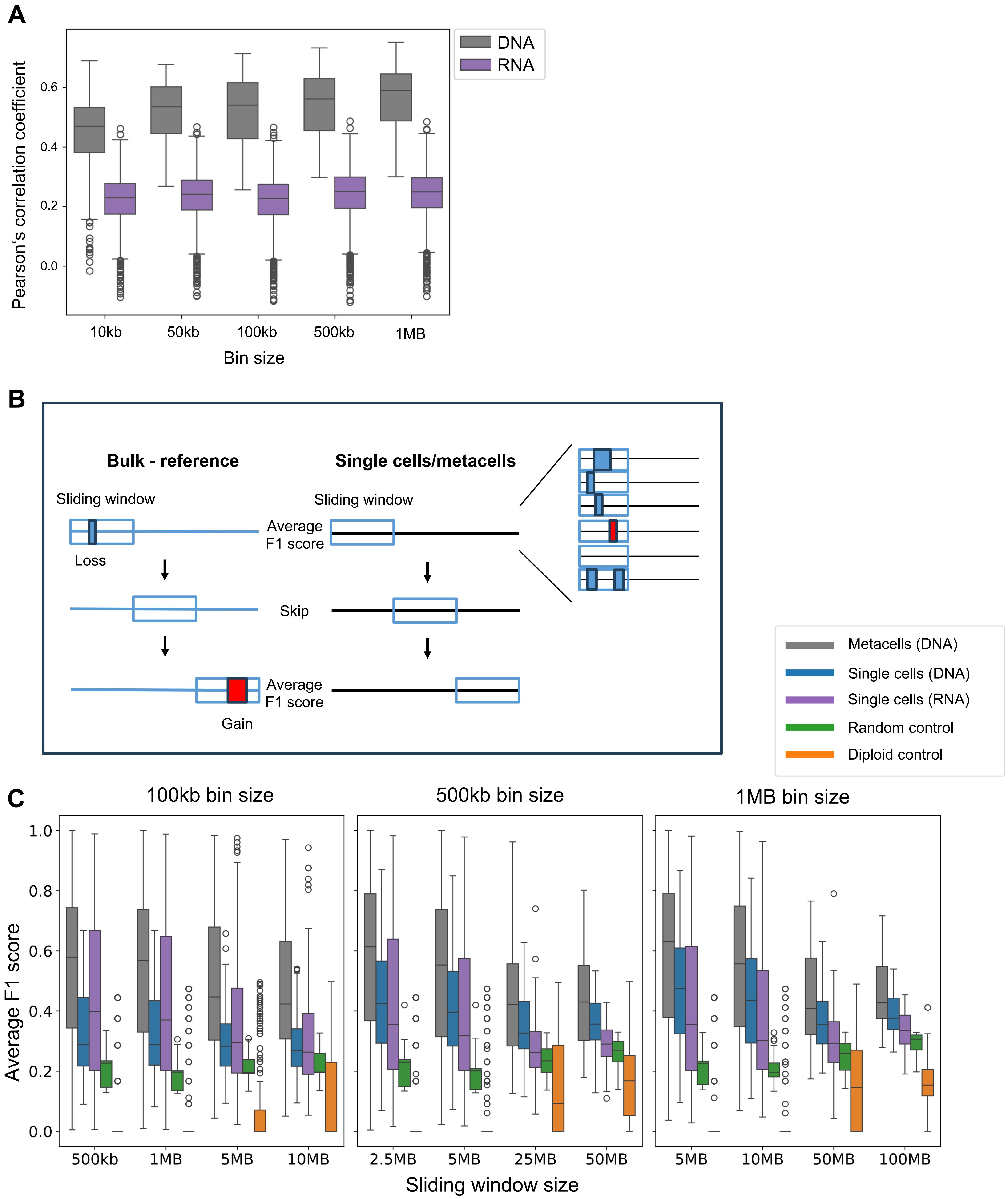

Fig. S6 | Resolution analysis of CNV estimates from DNA and RNA components of HIPSD&R-seq. (A) CNV estimates from metacells of DNA component of cluster 0 and cells of RNA component of cluster 0, both from figure 2, were correlated to CNV estimates of bulk WGS data of the Patient 87. Shown are Pearson's correlation coefficients for pairs of alternative methods with respect to bulk WGS. For each bin size, only genomic bins that overlap between both components of HIPSD&R-seq were used to calculate correlations. (**B**) Schematic overview of resolution analysis in **C**), where a non-overlapping sliding window with various sizes is applied to calculate an average F1 score between CNV estimates from single cells/metacells and CNV estimates for a reference bulk profile. An average F1 score across all cells/metacells is reported for a single window position. Windows that are fully diploid in bulk, are skipped. **C)** Average F1 score between CNV estimates of bulk WGS data of the Patient 87 and CNV estimates from metacells, single cells (DNA and RNA) of cluster 0, random control cells or fully diploid cells (see **Methods**) for bin sizes 100kb, 500kb and 1Mb. X axes represent the size of a sliding window.

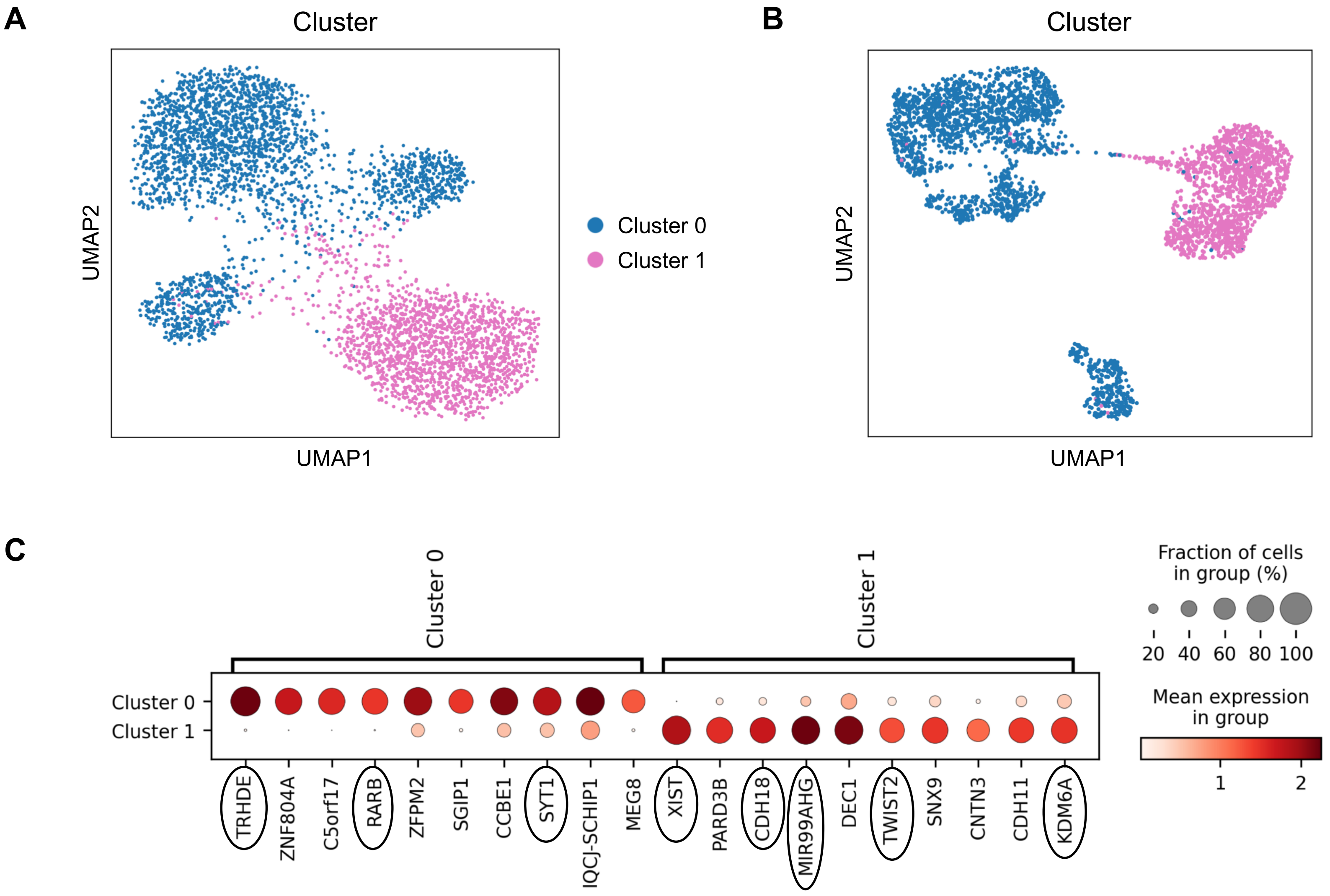

Fig. S7 | Analysis of differentially expressed genes between clusters in HIPSD&R-seq. UMAPs of scDNA-seq based on CNVs **(A)** and scRNA-seq based on gene expressions **(B)** (DNA and RNA components from HIPSD&R-seq). Points are colored based on unsupervised clustering of CNVs from scDNA-seq from fibroblasts from patient 41 and patient 87 mixed at a 1:1 ratio. Clusters remain well separated, showing that cells from patient 41 (cluster 1) are relatively homogenous at DNA and RNA level as compared to cells from patient 87, which comprise three major groups of cells retrieved at DNA level that also make three main groups of cells at RNA level. **(C)** Dotplot showing top 10 differentially expressed genes between cells from cluster 0 (correlated to male patient 87) and cluster 1 (correlated to female patient 41) from HIPSD&R-seq data. Sex specific genes (e.g. *XIST*) as well as genes located in chromosome regions for which the copy-number differs between both patients are among the top differentially expressed genes and are circled.

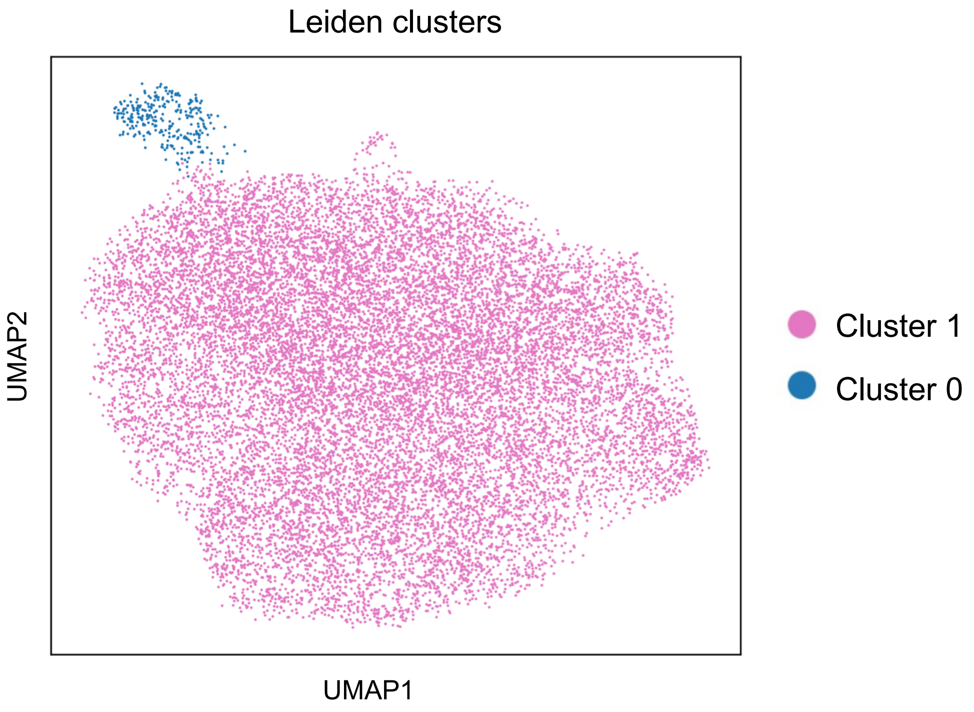

Fig. S8 | sciHIPSD-seq identifies a rare clone. UMAP with the two Leiden clusters from Figure 3. The mixed suspension with nuclei from both patients was analysed using sciHIPSD-seq and the 1% clone can be robustly identified by Leiden clustering of CNVs.

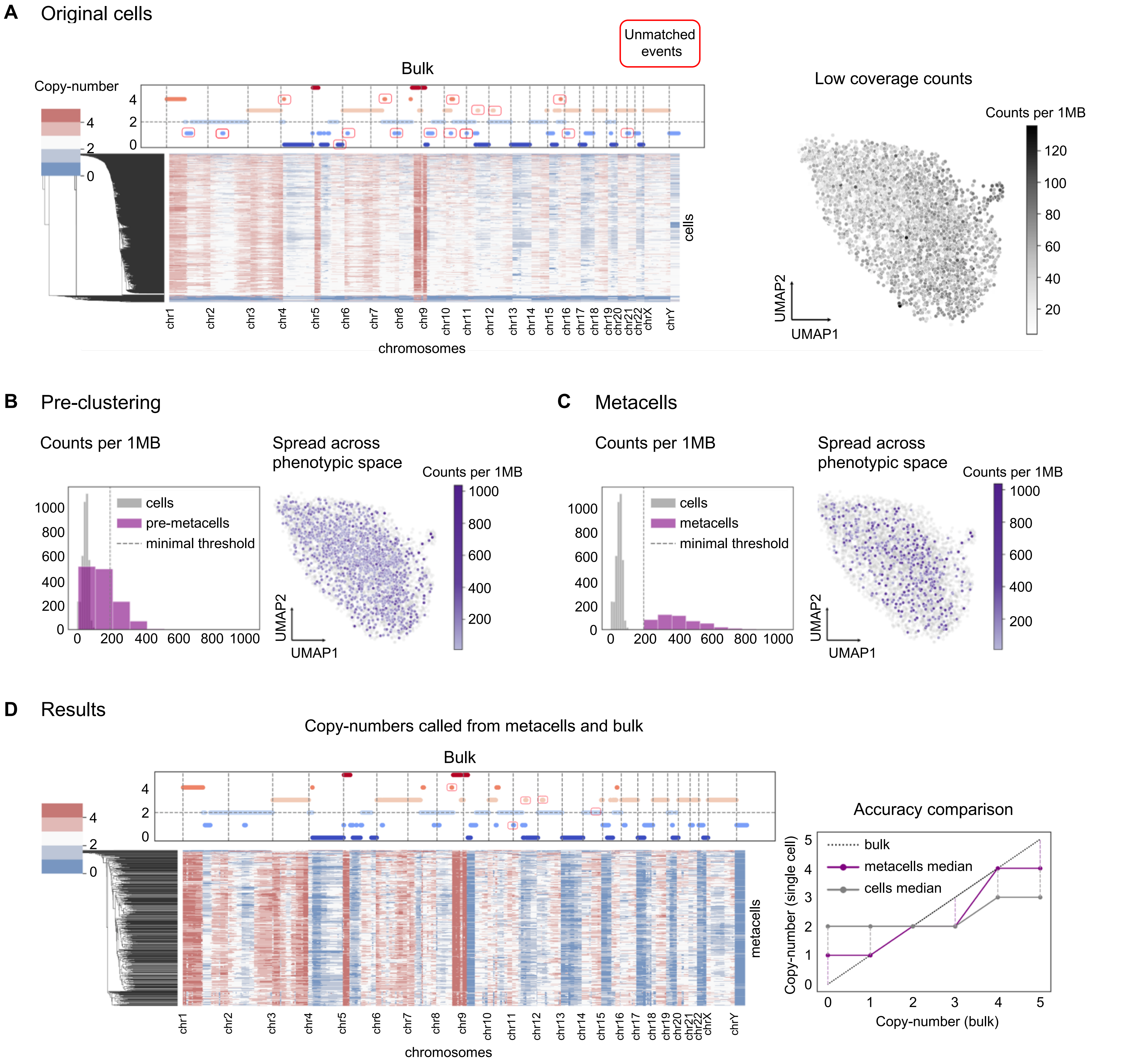

Fig. S9 | Metacelling workflow. **(A)** Pre metacelling results: copy number called on 100kb resolution for bulk data (top) and single cell (bottom); counts UMAP for 1MB window. **(B)** Cells are preclustered to show candidate metacells where some of the clusters might not have sufficient coverage. **(C)** Pre-metacells are greedily merged within a fixed distance until no metacell has a coverage lower than a selected threshold. (**D**) Copy number called on metacells (bottom) and corresponding bulk with fewer mismatches (top); comparison between bulk, initial cells and metacells shows that metacells produce closer match to bulk.
