# Supplementary material for: HIPSD&R-seq enables scalable genomic copy number and transcriptome profiling
Source: Genome Biol. 2024 Dec 18;25:316. doi: 10.1186/s13059-024-03450-0 (PMC11657747; doi:10.1186/s13059-024-03450-0)
Supplement: Supplementary file 3 — Additional file 3. Review history. [file 13059_2024_3450_MOESM3_ESM.docx]

Review History

**First round of review**

**Reviewer 1**

**Were you able to assess all statistics in the manuscript, including the appropriateness of statistical tests used?**

No

**Were you able to directly test the methods?**

No

**Comments to author:**

Lazareva et al. developed methods for scDNA-seq (HIPSD-seq) and single cell DNA + RNA-seq (HIPSD&R-seq) by modifying scATAC-seq and scMultiome released from 10x Genomics, respectively. First, they applied HIPSD-seq to human fibroblast cell line. HIPSD-seq showed quite lower coverage compared to the scCNV that is based on MDA (multiple displacement amplification) and used to be available from 10x Genomics. To reduce the limitation in coverage, they aggregated datasets of multiple cells with similar coverage distributions and called metacelling. They applied HIPSD&R-seq to PDX of medulloblastoma. They compared CNV patterns detected by HIPSD&R-seq with those by bulk WGS and scCNV. Finally, by applying combinatorial indexing to HIPSD-seq, they developed sciHIPSD&R-seq and enhanced the number of cells which can be analyzed at once. They showed minor cell population (~1%) can be detected by sciHIPSD&R-seq.
This method would be of interest to many researchers, but its novelty is limited because similar method already exist. Therefore, in order to demonstrate the value of this method, it must be firmly compared to existing similar method. In addition, there is a lack of sufficient information and explanation making it difficult to evaluate the manuscript.

1. As described in the Background section of this manuscript, a preprint about DEFND-seq, a joint method of DNA-seq and RNA-seq from the same single cells using 10x Chromium system was deposited in Biorxiv. This method is quite similar with HIPSD&R-seq. Therefore, the authors must demonstrate superior performance of HIPSD&R-seq to DEFND-seq. For the purpose, detailed comparison with DEFEND in performance would be essential.

Ref. https://www.biorxiv.org/content/10.1101/2023.02.09.527940v1.full.pdf

2. Although there is the section of "HIPSD&R-seq from fibroblasts" in the Methods, I could not find the dataset of HIPSD&R-seq from fibroblasts in Results section and Supplementary Table 1. If the dataset of HIPSD&R-seq from fibroblasts is not used in this study, they should remove the "HIPSD&R-seq from fibroblasts" section from the manuscript.

3. In Figure 2, the authors showed CNV patterns estimated from bulk DNA-seq. However, description about data analysis of bulk DNA-seq does not exist in the Methods section. The description should be included in the manuscript.

4. Although patients-derived samples were used, we could not find ethical information. Even if they used cell suspension prepared in their previous study, ethical information should be included also in this manuscript.

5. Colors used for HIPSD&R-seq and 10x DNA in plots of Supplementary Figure 1 are difficult to distinguish for people with color blindness.

6. Fundamental sequencing stats, such as total reads and median reads per cell before deduplication were not included.

7. Since the methods are modified versions of scATAC and scMultiome, there is a possibility that ATAC-dependent bias remains. Therefore, it is necessary to evaluate whether the pattern observed in scATAC (periodicity of insert length derived from nucleosomes, read concentration at open chromatin regions (such as TSS enrichment)) has disappeared.

8. To evaluate leak of DNA fragments from nuclei, I would recommend mixed species validation.

9. Information about reference genome and transcripts used in this study should be included.

10. The version of tools used in this study should be described.

11. Although high-concentrated Tn5 transposase was used in the methods, details of the transposase, such as origin and preparation method, were undescribed. They must include the detail of the transposase.

12. As for HIPSD&R-seq of PDX, if present, subpopulations with different CNV patterns and their characteristics in RNA expression should also be analyzed.

13. As for Supplementary Table 1, it is unclear whether median counts per cell is before or after removal of duplicate.

14. The duplicate rate varies so much among datasets of HIPSD&R-seq, HIPSD&R-seq, and sciHIPSD-seq. It seems that duplicate rates do not correlate with median count per cells.

15. Overall, it would be easier to understand if the explanations of figures are enriched.

**Reviewer 2**

**Were you able to assess all statistics in the manuscript, including the appropriateness of statistical tests used?**

No

**Were you able to directly test the methods?**

No

**Comments to author:**

The Lazareva et al. manuscript developed an assay (HIPSD&R-seq) to profile CNVs and transcriptomes of the same single cells in a high-throughput manner. The key step of the approach is to employ a nucleosome depletion, so the authors can apply commercially available single ATAC+RNA dual omics platforms to achieve simultaneous capture of genomic DNA and RNA of the same cell. While this strategy is interesting, the current version of the manuscript lacked adequate benchmarking for its robustness and reliability in CNV calling. Besides, the manuscript made little effort to demonstrate the application of the transcriptome part. Please find my concerns below.

Major concerns and suggestions:
1. The presented results strongly suggest a systematic bias of genome coverage. In Fig. 2A, it is clear to see that the aggregated single cells do not match the bulk data. For example, chr2q has an integer copy number = 2 in bulk, but the number is predominantly 3 in single cells. A more obvious example is chr15, where bulk copy number =3, but single cells are 1 or 2. Consistent with this observation, the overall correlation of single cell calls versus bulk calls is low (below 0.7, Fig. 2B).

I am wondering whether the bias is due to inefficient nucleosome depletion. To examine this, the authors may compare the "CNV" patterns to the ATAC peaks (or A/B genome compartments) of the same cell to examine the level of residual biased sampling due to different levels of genome accessibility. The authors also probably need to improve the nucleosome depletion conditions for more accurate CNV calling.

2. In Supp. Fig. 1G, the coverage of sciHIPSD-seq showed large variations among single cells. It is unclear whether it is due to nucleosome depletion efficiency or other factors. If it is the former case, with the potential different levels of bias contributed by ATAC fragments in different single cells, the metacell approach becomes concerning. It is possible that the "genomic similarity" the authors believed in could be totally epigenomic similarity. One way to refute the point is to split the cells into multiple groups based on coverage, and then examine the aggregated CNV patterns of different groups. (This is kind of what the authors presented in Supp. Fig. 2A, but please provide direct statistics.)

3. The benchmark analysis of the manuscript focused on the genome, leaving the transcriptome almost completely ignored, except for the very basic metric of gene detection, and transcriptome-inferred CNV. I will suggest to the authors to provide at least one showcase to demonstrate their transcriptome could reveal cellular heterogeneity at its sensitivity, for example, can they identify different cell states (for example cell cycle in the cell line, or different clusters in the PDX) out of the transcriptome, more interestingly, what extent can the transcriptomic heterogeneity be explained by genomic heterogeneity?


Minor points:
1. For better clarity, please refer to the specific figure panels in the text, rather than referring to "Supplementary Fig. 1" as a whole.
2. Fig. 3 scatterplot: please plot the grey and red dots in a random order, or add some transparency, so that the red dots are not completely overwritten by the grey ones.

**Authors Response**

**Point-by-point responses to the reviewers’ comments:**

**Point by point response to the comments from the reviewers**

Reviewer reports:

Reviewer #1:

Lazareva et al. developed methods for scDNA-seq (HIPSD-seq) and single cell DNA + RNA-seq (HIPSD&R-seq) by modifying scATAC-seq and scMultiome released from 10x Genomics, respectively. First, they applied HIPSD-seq to human fibroblast cell line. HIPSD-seq showed quite lower coverage compared to the scCNV that is based on MDA (multiple displacement amplification) and used to be available from 10x Genomics. To reduce the limitation in coverage, they aggregated datasets of multiple cells with similar coverage distributions and called metacelling. They applied HIPSD&R-seq to PDX of medulloblastoma. They compared CNV patterns detected by HIPSD&R-seq with those by bulk WGS and scCNV. Finally, by applying combinatorial indexing to HIPSD-seq, they developed sciHIPSD-seq and enhanced the number of cells which can be analyzed at once. They showed minor cell population (~1%) can be detected by sciHIPSD-seq.

This method would be of interest to many researchers, but its novelty is limited because similar method already exist. Therefore, in order to demonstrate the value of this method, it must be firmly compared to existing similar method. In addition, there is a lack of sufficient information and explanation making it difficult to evaluate the manuscript.

1. As described in the Background section of this manuscript, a preprint about DEFND-seq, a joint method of DNA-seq and RNA-seq from the same single cells using 10x Chromium system was deposited in Biorxiv. This method is quite similar with HIPSD&R-seq.  Therefore, the authors must demonstrate superior performance of HIPSD&R-seq to DEFND-seq. For the purpose, detailed comparison with DEFEND in performance would be essential.

Ref. <https://www.biorxiv.org/content/10.1101/2023.02.09.527940v1.full.pdf>

As suggested, we have directly compared DEFND-seq and HIPSD&R-seq, which includes a detailed evaluation of performance parameters of both methods for the DNA and RNA components.

For this comparison, we have applied both assays to the same source sample, composed of a 1:1 mix of fibroblasts from two patients. For DEFND-seq, we carefully reproduced the assay, closely following the protocol from Biorxiv, and used the version of the protocol that should perform the best according to the authors (e.g., nucleosome depletion using lithium diiodosalicylate assisted nucleosome depletion (LAND)). Both libraries were pooled and sequenced on three lanes on a NovaSeq 6000 S4 flowcell to enable a like with like comparison. We have also applied ATAC to the same sample (described in more detail in our response to point #7 below, comparison with ATAC).

*Comparison of the single-nucleus* ***DNA*** *sequencing between DEFND-seq and HIPSD&R-seq:*

HIPSD&R and DEFND are overall comparable in terms of canonical performance parameters (see details below), but HIPSD&R retrieves 6x more nuclei as compared to DEFND for the same amount of starting material:

HIPSD&R: 7847 cells defined after cutoff

DEFND: 1282 cells defined after cutoff

**Supplementary Figure 1 B-C.** Barcode rank plots showing all barcodes detected in comparative HIPSD&R-seq and DEFND-seq experiment for the DNA component. The blue lines show detected nuclei and the grey lines show the background. The input material was the same for both methods and the libraries were sequenced with the same coverage. The threshold for filtering nuclei is defined as the highest gradient in the barcode rank plot.

| **Method** | **Number of nuclei** | **Median of total reads per nucleus** | **Median of non-empty bins per nucleus (1MB)** | **Median TSS score enrichment on putative cells** |
| --- | --- | --- | --- | --- |
| HIPSD&R-seq | 7,847 | 49,233 | 2,826 | 1.88 |
| DEFND-seq | 1,282 | 623,644 | 2,920 | 1.10 |
| ATAC-seq | 20,000* | 41,262** | / | 3.41 |
| sciHIPSD-seq | 50,359 | 28,749 | 2,715 | 0.95 |

**Rebuttal Table 1.** **Comparison of performance parameters between HIPSD&R-seq and DEFND-seq.** The input material was the same for both methods (1:1 mix of fibroblasts from two patients) and the libraries were sequenced with the same coverage. Data for ATAC and sciHIPSDR-seq are also shown for comparison. ATAC was done on the same source sample as HIPSD&R-seq and DEFND-seq (the comparison with ATAC is described in more detail in our response to point #7 below, “comparison with ATAC”). sciHIPSD-seq data are from a previous run done on a 99:1 mix instead of 1:1 mix of fibroblasts. Note: the sequencing depth for ATAC and sciHIPSD-seq is different.

*The number of nuclei for ATAC is larger as compared to HIPSD&R because we loaded more nuclei for ATAC.

**For ATAC, we provide the number of high-quality fragments, not reads per cell.

We then added an additional filtering step based on the number of non-empty bins (at least 90% of 1MB bins should be non-empty for a cell to be kept for downstream analyses. The total number of genomic bins for 1MB bin size is 3,102):

| **Method** | **Number of nuclei after filtering** | **Median of total reads per nucleus after filtering** | **Median of non-empty bins per nucleus (1MB) after filtering** | **Median TSS score enrichment after filtering based on the number of non-empty bins** |
| --- | --- | --- | --- | --- |
| HIPSD&R-seq | 4,490 | 77,741 | 2,877 | 1.90 |
| DEFND-seq | 1,268 | 627,982.5 | 2,920 | 1.10 |
| ATAC*-seq | / | / | / | / |
| sciHIPSD-seq | 17,319 | 60,690 | 2,864 | 0.94 |

**Rebuttal Table 2.** Comparison of performance parameters between HIPSD&R-seq and DEFND-seq after filtering for nuclei with at least 90% of non-empty bins. The input material was the same for both methods and the libraries were sequenced with the same coverage.

* at least 90% of the bins non-empty is not a filtering step used for the ATAC data (see revised Methods).

**Effect of cell filtering on HIPSD&R-seq data:**

84% of the total reads in a sample are kept after filtering (shown below is the filtering on the HIPSD&R-seq dataset).

As an additional measurement of uniformity, the Lorenz curves show no major difference between DEFND, HIPSD&R-seq and sciHIPSD-seq:

**Supplementary Figure 3 I.** Lorenz curves to assess the uniformity of coverage in DEFND, HIPSD&R-seq and sciHIPSD-seq with and without filtering cells based on non-empty bins.

Importantly, HIPSD&R-seq shows a higher correlation with CNV profiles derived from matching bulk WGS of the sources samples as compared to DEFND, and the copy-number profiles of the 1:1 mix of fibroblasts from two different patients (patient 87 and patient 41) can be more clearly identified from the HIPSD&R-seq data (Supplementary Figure 1):

**Supplementary Figure 1. Comparison between DEFND-seq and HIPSD&R-seq (DNA components, for the same sample, 1:1 mix of fibroblasts from two patients). (D, E)** Correlations of CNVs from DEFND-seq (DNA component, panel D) and from HIPSD&R-seq (panel E) to bulk WGS, respectively. The correlation with bulk WGS is higher for HIPSD&R-seq as compared to DEFND. Unsupervised clustering was performed on CNVs to identify two clusters. **(F-I)** Heatmaps show copy-number variation for DEFND-seq and for HIPSD&R-seq for the same sample. Each row shows one single nucleus in G and I and one metacell in F and H. Hmmcopy was used (1MB bin size). Blue, pink, brown and orange labels are based on the unsupervised clustering shown in **D** and **E.** HIPSD&R-seq provides more accurate copy-number inference as compared to DEFND-seq.

**Rebuttal Figure** **1.** Scatterplots showing the correlation coefficient (Pearson’s correlation coefficient) per cell between single-cell and bulk WGS-derived CNV profiles as a function of read coverage per cell. Top: correlations for HIPSD&R-seq, the bottom panels show the correlations for DEFND-seq. Even though DEFND has more coverage, it does not replicate bulk as well as HIPSD&R.

*Comparison of the single-nucleus* ***RNA*** *sequencing between DEFND-seq and HIPSD&R-seq:*

The absence of a sharp knee in the barcode rank plots suggests ambient RNA contamination in DEFND, whereas the presence of a knee in HIPSD&R does not indicate any detectable ambient RNA contamination. Contamination fraction computed with SoupX further supports a higher contamination in DEFND as compared to HIPSD&R (Supplementary Figure 2 below).

This ambient RNA contamination in DEFND is potentially due to the fact that there is no fixation step before nucleosome depletion in the DEFND protocol, which can lead to leakage from nuclei. When we developed HIPSD&R and sciHIPSD, we decided to include a fixation step to ensure a high integrity of the nuclei and prevent this issue.

In addition, lithium diiodosalicylate (used in DEFND) not only disrupts DNA-protein interactions to generate nucleosome-free genomic DNA but also affects nuclear organization and pore complexes (PMID: 3696163). Furthermore, lithium diiodosalicylate and xSDS (fixation) were compared by Vitak et al (PMID: [28135258](https://pubmed.ncbi.nlm.nih.gov/28135258)) demonstrating a better performance in terms of genome coverage for xSDS. This is why we decided to use xSDS (and not lithium diiodosalicylate like in DEFND) for nucleosome depletion.

Notably, these signatures of ambient RNA contamination are also visible in the DEFND datasets generated by the authors of DEFND, which are publicly available, showing that this quality issue is not due to an experimental mistake when we perform DEFND:

Public DEFND data, reanalyzed by us:

1. Public DEFND_GEX - BJ fibroblasts ([SRR23292070](https://trace.ncbi.nlm.nih.gov/Traces?run=SRR23292070))

2. Public DEFND_GEX - GBM ([SRR23292060](https://trace.ncbi.nlm.nih.gov/Traces?run=SRR23292060)):

Our data **HIPSD&R_GEX**:

Our data **DEFND_GEX** (generated on the same sample as HIPSD&R for direct comparison):

**Supplementary Figure 2.** Left, Barcode rank plots showing all barcodes detected in public DEFND data (1. BJ fibroblasts, 2. glioblastoma) as well as in our own comparative HIPSD&R-seq and DEFND-seq experiment. The blue lines show detected nuclei and the grey lines show the background. For the comparative HIPSD&R-seq and DEFND-seq experiment, the input material was the same for both methods and the libraries were sequenced with the same depth. Right, contamination fraction computed with SoupX. The contamination fraction is shown on the x-axis, with 0 meaning no contamination and 1 meaning that 100% of UMIs in a droplet are soup.

| **Sample Name** | **Method** | **Estimated Number of Nuclei (total)** | **Mean Reads per Nucleus** | **Median counts per Nucleus** | **Median Genes per Nucleus (GRCh38)** | **Median % of mitochondrial counts per nucleus** | **Median % of ribosomal counts per nucleus** | **Valid Barcodes** |
| --- | --- | --- | --- | --- | --- | --- | --- | --- |
| HIPSD&R_GEX | HIPSD&R-seq | 8,764 | 58,133 | 3,890.5 | 1,990 | 1.99 | 14.3 | 93.1% |
| DEFND_GEX | DEFND-seq | 2,457 | 153,895 | 7,618 | 3,637 | 0.75 | 4.2 | 93.2% |

**Rebuttal Table 3.** Comparison of performance parameters between HIPSD&R-seq and DEFND-seq. The input material was the same for both methods and the libraries were sequenced with the same coverage.

For the RNA component, HIPSD&R retrieves 3.5x more nuclei as compared to DEFND for the same amount of starting material.

For other parameters, HIPSD&R and DEFND are comparable. The number of genes, mitochondrial and ribosomal counts are in the typical range for 10x RNAseq data. As HIPSD&R retrieves more nuclei, the number of reads per nucleus is lower and hence the number of genes per nucleus is slightly lower (and the fraction of mitochondrial and ribosomal genes slightly higher), which is expected due to the different number of reads per nucleus.

The number of nuclei with valid **DNA & RNA** barcode (non-background) is:

- HIPSD&R - 7436 cells
- DEFND - 1255 cells

The number of nuclei with valid **DNA & RNA** barcode after filtering for nuclei with at least 90% of non-empty genomic bins for DNA is:

- HIPSD&R - 4362 cells
- DEFND - 1244 cells

Hence, these analyses show that despite non-perfect uniformity, HIPSD&R and sciHIPSD perform at least as well as DEFND, and provide a much higher throughput as compared to DEFND.

2. Although there is the section of "HIPSD&R-seq from fibroblasts" in the Methods, I could not find the dataset of HIPSD&R-seq from fibroblasts in Results section and Supplementary Table 1. If the dataset of HIPSD&R-seq from fibroblasts is not used in this study, they should remove the "HIPSD&R-seq from fibroblasts" section from the manuscript.

We have now added all datasets mentioned in the revised Results section to revised Supplementary Table 1.

3. In Figure 2, the authors showed CNV patterns estimated from bulk DNA-seq. However, description about data analysis of bulk DNA-seq does not exist in the Methods section. The description should be included in the manuscript.

Thanks for pointing this out. We have now added the missing Methods section on the data analysis of bulk DNA-seq to the revised version of the manuscript (please see Methods, “Analysis of bulk whole-genome sequencing data”).

4. Although patients-derived samples were used, we could not find ethical information. Even if they used cell suspension prepared in their previous study, ethical information should be included also in this manuscript.

Thanks for pointing this out. We have now added the ethical information to the revised version of the manuscript (please see “Ethics approval and consent to participate”).

5. Colors used for HIPSD&R-seq and 10x DNA in plots of Supplementary Figure 1 are difficult to distinguish for people with color blindness.

We apologize for this issue, in the revised version of the manuscript we have tried to use color codes that are suitable with color blindness.

6. Fundamental sequencing stats, such as total reads and median reads per cell before deduplication were not included.

We have now added the total number of reads and median number of reads per cell before deduplication to the revised version of the manuscript (see revised Supplementary Table 1).

7. Since the methods are modified versions of scATAC and scMultiome, there is a possibility that ATAC-dependent bias remains. Therefore, it is necessary to evaluate whether the pattern observed in scATAC (periodicity of insert length derived from nucleosomes, read concentration at open chromatin regions (such as TSS enrichment)) has disappeared.

As suggested, we have evaluated TSS enrichment and periodicity to see whether patterns observed in scATAC had disappeared. For this comparison, we performed ATAC on the same sample as HIPSD&R. We used Epianeufinder to obtain CNV profiles from ATAC. These analyses are shown in new Supplementary Figure 5.

Whereas in ATAC the TSS score is around 3.4, as expected for ATAC data, the TSS score for HIPSD&R-seq and for sciHIPSD are below 2 and below 1, respectively, showing that no significant ATAC-dependent bias remains.

The minimal signal at TSS in HIPSD&R-seq is due to the fact that we included a fixation step to ensure a high integrity of the nuclei and prevent ambient RNA contamination (see response to point #1 above, the ambient RNA contamination in DEFND is potentially due to the fact that there is no fixation step before nucleosome depletion in the DEFND protocol, which can lead to leakage from nuclei).

| **Method** | **Number of nuclei** | **Median of total reads per cell** | **Median of non-empty bins per cell (1MB)** | **Median TSS score enrichment** |
| --- | --- | --- | --- | --- |
| HIPSD&R-seq | 7,847 | 49,233 | 2,826 | 1.88 |
| ATAC-seq | >20,000* | 41,262** | / | 3.41 |
| sciHIPSD-seq | 50,359 | 28,749 | 2,715 | 0.95 |

**Supplementary Figure 5A.** Comparison of TSS score enrichment between HIPSD&R-seq, ATAC-seq and sciHIPSD-seq. The input material was the same sample for HIPSD&R-seq and ATAC-seq. The sciHIPSD-seq data are from a previous run with a different ratio of the fibroblasts from the same two patients (99:1 for sciHIPSD instead of 50:50 for HIPSD&R and ATAC).

*The number of nuclei for ATAC is larger as compared to HIPSD&R because we loaded more nuclei for ATAC.

**For ATAC, we provide the number of high-quality fragments, not reads per cell.

The fragment size distribution of ATAC-seq has a clear periodicity, as expected (**Supplementary Figure 5B below**). Importantly, this periodicity is not observed in HIPSD&R or sciHIPSD, showing no ATAC-dependent bias:

Fragment length (bp):

**Supplementary Figure 5B.** Fragment length distribution for ATAC-seq has a clear periodicity, as expected. However, this periodicity is not observed in HIPSD&R or sciHIPSD-seq data, showing no ATAC-dependent bias.

Importantly, the correlation with bulk WGS is much higher for HIPSD&R-seq as compared to copy-number inference from scATAC-seq:

**Supplementary Figure 5E-F.** Correlation of CNVs from HIPSD&R-seq (DNA component, left) and from scATAC-seq (right) to bulk WGS, respectively. Unsupervised clustering was performed on CNVs to identify two clusters.

In addition, HIPSD&R-seq (top heatmap) provides much more accurate copy-number inference as compared to ATAC-seq (bottom heatmap):

**Supplementary Figure 5C-D.** Heatmaps show copy-number variation for HIPSD&R-seq on the top and ATAC-seq on the bottom for the same sample (1:1 mix of fibroblasts from two patients). Each row shows one single nucleus. For ATAC-seq, Epianeufinder was used and for HIPSD&R-seq Hmmcopy was used (1MB bin size). Blue and pink labels are based on the unsupervised clustering above.

Collectively, our analyses show that despite non-perfect uniformity, HIPSD&R and sciHIPSD perform better than ATAC and DEFND for copy-number analysis.

8. To evaluate leak of DNA fragments from nuclei, I would recommend mixed species validation.

We have performed two types of analyses to address this question.

First, we analysed a mix of human blood cells with mouse cells (ratio of 3:1). We present the results in Rebuttal Figure 2 below.

We aligned the reads from the mixed species experiment to the cellranger’s concatenated human + mouse genome and then classified nuclei as either human, mouse or mixed based on the percentage of reads per nucleus aligned to each genome. If at least 90% of the reads per barcode were aligned to a human genome, the nucleus was classified as human. If at least 90% of the reads per barcode were aligned to a mouse genome, the nucleus was classified as mouse. For all other cases, the nucleus was classified as mixed (modified approach from <https://kb.10xgenomics.com/hc/en-us/articles/115003517183-How-does-cellranger-count-identify-multiplets>).

The combined percentage of multiplets is below 5% and thus is in the expected range for 8000 recovered cells. Furthermore, nuclei assigned to human and mouse show a clear separation along the x and y-axis, respectively.

| **Human** | **Mouse** | **Mixed** | **Total** |
| --- | --- | --- | --- |
| 6253 | 1158 | 353 | 7764 |

**Rebuttal Figure 2.** Mixed species validation using human blood cells and mouse cells.

However, this mixed species experiment does not formally rule out leakage from nuclei.

Hence, we have performed additional experimental validation by brightfield microscopy and shown that the nuclei look visually intact (left side, positive control for a lysed nucleus, right side, intact nucleus).

Hence, even though careful monitoring of the integrity of the nuclei and adjustments to each sample type are indeed important and required for these methods, these analyses show that leakage of DNA fragments from nuclei is not a major concern for our assay.

9. Information about reference genome and transcripts used in this study should be included.

We have now added information about the reference genome GRCh38 and transcripts used in this study (see revised Methods, “Computational analyses”).

10. The version of tools used in this study should be described.

We have now added the version of all tools used in this study (see revised Methods).

11. Although high-concentrated Tn5 transposase was used in the methods, details of the transposase, such as origin and preparation method, were undescribed. They must include the detail of the transposase.

We now refer to methods from “Enhancing sensitivity and versatility of Tn5-based single cell omics”, Seufert et al.

# “Expression and purification of the Tn5 transposase

Purified unloaded Tn5 was obtained from the EMBL Protein Expression and Purification Core Facility, which was produced and stored as described previously ([Hennig et al., 2018](https://www.frontiersin.org/articles/10.3389/freae.2023.1245879/full#B14)) with some adjustments resulting in the following workflow: The pETM11-Sumo3-Tn5 plasmid encoding His6-Sumo3-tagged Tn5 E54K L372P was freshly transformed into *E. coli* BL21(DE3) codon + RIL cells (Stratagene). Precultures were grown overnight at 37°C in LB medium supplemented with 30 µg/ml kanamycin and 34 µg/ml chloramphenicol and used to inoculate the large-scale expression cultures. A 10 ml preculture was added to 1 liter of TB-FB medium supplemented with 2 mM MgSO4, 0.05% glucose, 1.5% lactose, 30 µg/ml kanamycin and 34 µg/ml chloramphenicol. The cultures were grown at 37°C until the OD600 reached −0.6, after which the temperature was reduced to at 18°C. After overnight expression at 18°C, the cultures were harvested by centrifugation (30 min, 5000 x *g*, 4°C) and the pellets were flash-frozen in liquid nitrogen and stored at −80°C until the start of the protein purification.

For the protein purification, all chromatography steps were performed at 4°C and samples were kept on ice at all times. The cell pellet was resuspended in cold running buffer (20 mM Hepes-NaOH pH 7.2, 800 mM NaCl, 5 mM imidazole, 1 mM EDTA, 2 mM DTT and 10% glycerol) supplemented with cOmplete EDTA-free protease inhibitors (Roche). The cells were lysed by 5 passages through a microfluidizer, followed by centrifugation (30 min, 140000 x *g*, 4°C). After centrifugation, a 10% polyethyleneimine (PEI) solution at pH 7.2 was added dropwise to the cleared lysate to a final concentration of 0.5% while constantly stirring the solution. To remove the PEI-precipitated fraction, the lysate was subjected to another centrifugation step at 20000 x *g* for 30 minutes at 4°C. The cleared lysate containing the recombinant His6-Sumo3-tagged Tn5 protein was then loaded on a 5 ml prepacked cOmplete His-tag purification column (Roche) equilibrated in running buffer. After loading the sample, the cOmplete column was washed with the same buffer until the absorbance signal at 280 nm returned to baseline. The column was eluted with a 1-step elution in running buffer supplemented with 300 mM imidazole. The elution fractions were analysed via SDS-PAGE and the fractions containing the His6-Sumo3-tagged Tn5 were pooled and dialysed overnight to running buffer in the presence of His6-tagged SenP2 protease to remove the His6-Sumo3 fusion tag. The next morning, the dialysed sample was loaded again on a 5 ml cOmplete His-tag purification column equilibrated with running buffer. The untagged Tn5 was collected in the flow through of the column and further purified using size exclusion chromatography. The Tn5 sample was loaded on a HiLoad Superdex 200 16/600 pg column (GE Healthcare) equilibrated with 50 mM Tris pH 7.5, 800 mM NaCl, 0.2 mM EDTA, 2 mM DTT and 10% glycerol. The elution fractions were analysed via SDS-PAGE and the dimer peak fractions were pooled. The final Tn5 sample was aliquoted and stored at −20°C in 25 mM Tris pH 7.5, 800 mM NaCl, 0.1 mM EDTA, 1 mM DTT and 50% glycerol at a concentration of 1 mg/ml.”

12. As for HIPSD&R-seq of PDX, if present, subpopulations with different CNV patterns and their characteristics in RNA expression should also be analyzed.

To address this question, we have used a newly generated HIPSD&R-seq dataset of fibroblasts mixed from a male and a female patients at a 1:1 ratio. We have performed proof of concept differential expression analysis between these patient-related clones and showed that expected differences in expression can be detected. Specifically, we have identified canonical sex-specific genes (e.g. *XIST*) as well as genes located in chromosome regions for which the copy-number differs between both patients belong to the most differentially expressed genes. These analyses are shown in new Supplementary Figure 7.

**Supplementary Figure 7A-B: UMAPs of scDNA-seq (left) and scRNA-seq (right) (DNA and RNA components from HIPSD&R-seq).** Points are colored based on unsupervised clustering of CNVs from scDNA-seq from fibroblasts from patient 41 and patient 87 mixed at a 1:1 ratio.

**Supplementary Figure 7C: Dotplot showing top differentially expressed genes between cells from cluster 0 (correlated to male patient 87) and cluster 1 (correlated to female patient 41) in HIPSD&R-seq data.** Sex specific genes (e.g. *XIST*) as well as genes located in chromosome regions for which the copy-number differs between both patients belong to the most differentially expressed genes and are circled.

13. As for Supplementary Table 1, it is unclear whether median counts per cell is before or after removal of duplicate.

We apologize for the lack of clarity. The median counts per cell are after removal of duplicates. We have now added this information to the revised version of the manuscript.

14. The duplicate rate varies so much among datasets of HIPSD&R-seq, HIPSD&R-seq, and sciHIPSD-seq. It seems that duplicate rates do not correlate with median count per cell.

| **Method** | **Total number of read pairs** | **Median percentage of duplicates per cell** |
| --- | --- | --- |
| HIPSD&R-seq | 1,445,045,028 | 90.58% |
| sciHIPSD-seq | 4,603,867,891 | 32.73% |

It is correct that duplicate rates do not correlate with median count per cell. We reach a higher library complexity with sciHIPSD as compared to HIPSD&R-seq. HIPSD&R-seq uses the multiome chemistry from 10X that relies on a ligation step to barcode DNA molecules that are purified afterwards without prior amplification. For sciHIPSD, amplified barcoded genomic DNA is generated in droplets prior to purification. Thus the complexity for the DNA-only readout is expected to show higher library complexity in general.

We have also seen that the duplication rate varies between DEFND and HIPSD&R. This is most likely due to the fixation that we use for HIPSD&R. Fixation in itself supports nuclei integrity (as shown above, please kindly refer to Suppl. Fig. 2 and response to point #8) and also results in a more uniform read distribution, as shown by others (PMID: [28135258](https://pubmed.ncbi.nlm.nih.gov/28135258)). Thus, even with a higher duplication rate, we achieve better resolution due to (i) uniform coverage and (ii) preservation of nuclei integrity.

15. Overall, it would be easier to understand if the explanations of figures are enriched.

As suggested, we have added more information to the revised figure captions to improve clarity.

Reviewer #2:

The Lazareva et al. manuscript developed an assay (HIPSD&R-seq) to profile CNVs and transcriptomes of the same single cells in a high-throughput manner. The key step of the approach is to employ a nucleosome depletion, so the authors can apply commercially available single ATAC+RNA dual omics platforms to achieve simultaneous capture of genomic DNA and RNA of the same cell. While this strategy is interesting, the current version of the manuscript lacked adequate benchmarking for its robustness and reliability in CNV calling. Besides, the manuscript made little effort to demonstrate the application of the transcriptome part. Please find my concerns below.

Major concerns and suggestions:

1. The presented results strongly suggest a systematic bias of genome coverage. In Fig. 2A, it is clear to see that the aggregated single cells do not match the bulk data. For example, chr2q has an integer copy number = 2 in bulk, but the number is predominantly 3 in single cells. A more obvious example is chr15, where bulk copy number =3, but single cells are 1 or 2. Consistent with this observation, the overall correlation of single cell calls versus bulk calls is low (below 0.7, Fig. 2B).

I am wondering whether the bias is due to inefficient nucleosome depletion. To examine this, the authors may compare the "CNV" patterns to the ATAC peaks (or A/B genome compartments) of the same cell to examine the level of residual biased sampling due to different levels of genome accessibility. The authors also probably need to improve the nucleosome depletion conditions for more accurate CNV calling.

Please also kindly refer to point #7 from Reviewer 1.

As suggested, we have evaluated TSS enrichment and periodicity to see whether patterns observed in scATAC had disappeared. For this comparison, we performed ATAC on the same sample as HIPSD&R. We used Epianeufinder to obtain CNV profiles from ATAC. These analyses are shown in new Supplementary Figure 5.

Whereas in ATAC the TSS score is around 3.4, as expected for ATAC data, the TSS score for HIPSD&R-seq and for sciHIPSD are below 2 and below 1, respectively, showing that no significant ATAC-dependent bias remains. The minimal signal at TSS in HIPSD&R-seq is due to the fact that we included a fixation step to ensure a high integrity of the nuclei and prevent ambient RNA contamination (see response to point #1 above, the ambient RNA contamination in DEFND is potentially due to the fact that there is no fixation step before nucleosome depletion in the DEFND protocol, which can lead to leakage from nuclei).

| **Method** | **Number of nuclei** | **Median of total reads per cell** | **Median of non-empty bins per cell (1MB)** | **Median TSS score enrichment** |
| --- | --- | --- | --- | --- |
| HIPSD&R-seq | 7,847 | 49,233 | 2,826 | 1.88 |
| ATAC-seq | >20,000* | 41,262** | / | 3.41 |
| sciHIPSD-seq | 50,359 | 28,749 | 2,715 | 0.95 |

**Supplementary Figure 5A.** Comparison of TSS score enrichment between HIPSD&R-seq, ATAC-seq and sciHIPSD-seq. The input material was the same sample for HIPSD&R-seq and ATAC-seq. The sciHIPSD-seq data are from a previous run with a different ratio of the fibroblasts from the same two patients (99:1 for sciHIPSD instead of 50:50 for HIPSD&R and ATAC).

*The number of nuclei for ATAC is larger as compared to HIPSD&R because we loaded more nuclei for ATAC.

**For ATAC, we provide the number of high-quality fragments, not reads per cell.

The fragment size distribution of ATAC-seq has a clear periodicity, as expected (**Supplementary Figure 5B below**). Importantly, this periodicity is not observed in HIPSD&R or sciHIPSD, showing no ATAC-dependent bias:

Fragment length (bp):

**Supplementary Figure 5B.** Fragment length distribution for ATAC-seq has a clear periodicity, as expected. However, this periodicity is not observed in HIPSD&R or sciHIPSD-seq data, showing no ATAC-dependent bias.

Importantly, the correlation with bulk WGS is much higher for HIPSD&R-seq as compared to copy-number inference from scATAC-seq:

**Supplementary Figure 5E-F.** Correlation of CNVs from HIPSD&R-seq (DNA component, left) and from scATAC-seq (right) to bulk WGS, respectively. Unsupervised clustering was performed on CNVs to identify two clusters.

In addition, HIPSD&R-seq (top heatmap) provides much more accurate copy-number inference as compared to ATAC-seq (bottom heatmap):

**Supplementary Figure 5C-D.** Heatmaps show copy-number variation for HIPSD&R-seq on the top and ATAC-seq on the bottom for the same sample (1:1 mix of fibroblasts from two patients). Each row shows one single nucleus. For ATAC-seq, Epianeufinder was used and for HIPSD&R-seq Hmmcopy was used (1MB bin size). Blue and pink labels are based on the unsupervised clustering above.

Collectively, our analyses show that despite non-perfect uniformity, HIPSD&R and sciHIPSD perform better than ATAC for copy-number analysis.

2. In Supp. Fig. 1G, the coverage of sciHIPSD-seq showed large variations among single cells. It is unclear whether it is due to nucleosome depletion efficiency or other factors. If it is the former case, with the potential different levels of bias contributed by ATAC fragments in different single cells, the metacell approach becomes concerning. It is possible that the "genomic similarity" the authors believed in could be totally epigenomic similarity. One way to refute the point is to split the cells into multiple groups based on coverage, and then examine the aggregated CNV patterns of different groups. (This is kind of what the authors presented in Supp. Fig. 2A, but please provide direct statistics.)

For sciHIPSD, the high cell concentration used results in either one or two cells captured in one droplet (with doublets distinguished thanks to the barcodes) which in turn can cause coverage differences across single cells. The same amount of reagents per droplet, no matter whether one or more nuclei are contained in the droplet, leads to a broader coverage distribution, as described by others in the context of combinatorial indexing (see for instance PMID: 28135258, PMID: [34059827](https://pubmed.ncbi.nlm.nih.gov/34059827): broader z-score distribution of UMIs per cell for droplets with more than one cell). Even though the two (or more) nuclei can be analyzed separately due to the combinatorial barcoding, variations in coverage between nuclei can be expected.

To address if (i) cells cluster according to coverage or (ii) coverage depends on nucleosome depletion efficiency, we now provide additional analyses (Rebuttal Figures 3 to 6 below) showing that neither coverage nor TSS score contribute significantly to the separation of clusters. In addition, the total number of fragments per cell does not correlate with the TSS score as metrics for coverage and nucleosome depletion efficiency, respectively.

**Rebuttal Figure 3.** Leiden clustering on CNVs for sciHIPSD-seq, Number of non-empty bins per nucleus, number of total reads per nucleus and TSS score enrichment. The clusters are not driven by the TSS score enrichment or by the coverage.

**Rebuttal Figure 4.** The TSS score enrichment (as a surrogate for nucleosome depletion efficiency) converges towards 1 and we do not observe any link between TSS score enrichment and the total number of reads per nucleus.

**Rebuttal Figure 5.** Violin plots showing the number of reads per cell and the TSS score enrichment for each cluster in sciHIPSD-seq data from fibroblasts. Violin plots show a similar distribution of the number of reads per cell across clusters and a similar distribution of TSS score enrichment (used as a surrogate for the efficiency of nucleosome depletion).

**Rebuttal figure 6.** Heatmap showing CNVs from sciHIPSD-seq. The annotations on the right show cluster identity, number of non-empty bins (the total number of bins is 3102 for 1MB bin size) and TSS score enrichment. The non-empty bin colour coding along the sciHIPSD heatmap shows that the clones do not appear to be driven by the coverage.

However, even though metacelling has been very successful in the context of RNA studies, we do agree that metacelling is not always required for our workflow, and in fact we have now analysed sciHIPSD-seq data without metacelling, and shown a high correlation with bulk WGS (see revised Figures 2 and 3). In the revised version of the manuscript, we provide both analysis workflow options, namely without metacelling (e.g. potentially for applications to samples with very high genetic diversity) or with metacelling (e.g. potentially in the context of lower genetic diversity and/or lower coverage).

3. The benchmark analysis of the manuscript focused on the genome, leaving the transcriptome almost completely ignored, except for the very basic metric of gene detection, and transcriptome-inferred CNV. I will suggest to the authors to provide at least one showcase to demonstrate their transcriptome could reveal cellular heterogeneity at its sensitivity, for example, can they identify different cell states (for example cell cycle in the cell line, or different clusters in the PDX) out of the transcriptome, more interestingly, what extent can the transcriptomic heterogeneity be explained by genomic heterogeneity?

Please also kindly refer to point #12 from Reviewer 1.

To address this question, we have used a newly generated HIPSD&R-seq dataset of fibroblasts mixed from a male and a female patients at a 1:1 ratio. We have performed proof of concept differential expression analysis between these patient-related clones and showed that expected differences in expression can be detected. Specifically, we have identified canonical sex-specific genes (e.g. *XIST*) as well as genes located in chromosome regions for which the copy-number differs between both patients belong to the most differentially expressed genes. These analyses are shown in new Supplementary Figure 7.

**Supplementary Figure 7A-B: UMAPs of scDNA-seq (left) and scRNA-seq (right) (DNA and RNA components from HIPSD&R-seq).** Points are colored based on unsupervised clustering of CNVs from scDNA-seq from fibroblasts from patient 41 and patient 87 mixed at a 1:1 ratio.

**Supplementary Figure 7C: Dotplot showing top differentially expressed genes between cells from cluster 0 (correlated to male patient 87) and cluster 1 (correlated to female patient 41) in HIPSD&R-seq data.** Sex specific genes (e.g. *XIST*) as well as genes located in chromosome regions for which the copy-number differs between both patients belong to the most differentially expressed genes and are circled.

Minor points:

1. For better clarity, please refer to the specific figure panels in the text, rather than referring to "Supplementary Fig. 1" as a whole.

As suggested, we now refer to specific figure panels throughout the text rather than to entire figures.
2. Fig. 3 scatterplot: please plot the grey and red dots in a random order, or add some transparency, so that the red dots are not completely overwritten by the grey ones.

We have changed the color code and presentation in revised Figure 3 to improve clarity.

**Second round of review**

**Reviewer 1**

To address our comments, the authors conducted a comprehensive set of additional experiments and data analyses. I think they have addressed most of the comments. Especially, they were able to demonstrate several advantages of HIPSD&R-seq over DEFND-seq, such as lower contamination from ambient RNA, a higher number of cells (nuclei), and higher correlation with bulk DNA-seq, although it should be noted that the data from HIPSD&R-seq is significantly sparser than that from DEFND-seq.

**Reviewer 2**

Otonicar et al. have significantly improved the manuscript with the following components: 1) additional analysis of CNV calling accuracy and robustness; 2) additional analysis of transcriptome; and most importantly, 3) benchmark comparison against a similar method DEFEND and demonstrated the better performance. These additional analysis results highlighted the power of HIPSD&R in identifying rare CNV-defined populations in cell mixtures of different origins. I would like to suggest two changes in wording before fully acceptance. And additional review is not needed.

1. The authors showed the minimal TSS enrichment score and the lack of nucleosome periodicity patterns in HIPSD dataset, which suggested that the nucleosomes were efficiently depleted so that their impact on CNV inference was minimal. By quantifying the correlation with bulk CNV, the authors further concluded that the HIPSD&R DNA outperformed scRNA-seq or scATAC-seq. But the Pearson’s correlation coefficient compared to bulk is around 0.6 at 1MB resolution. This correlation should warrant robust clustering analysis, but probably not high enough to be stated as “accurate CNV calling”. I will probably state it as robust CNV calling. Consistent with this thought, the results of “metacell” CNV does have some deviations from bulk CNV calling in Supplementary Fig. 9, as mentioned in my original comments. I guess that it indicates that there are still some regional biases at 1MB resolution. Just out of curiosity, will these deviations be minimized if enlarging the binning windows, for both single cells and metacells?


2. I would like to suggest that the authors to provide more detailed descriptions when referring to Supplementary Figs. 1-3. and maybe provide a summary of the results in the main text.

**Authors Response**

**Point-by-point responses to the reviewers’ comments:**

**Reviewer #1:** To address our comments, the authors conducted a comprehensive set of additional experiments and data analyses. I think they have addressed most of the comments. Especially, they were able to demonstrate several advantages of HIPSD&R-seq over DEFND-seq, such as lower contamination from ambient RNA, a higher number of cells (nuclei), and higher correlation with bulk DNA-seq, although it should be noted that the data from HIPSD&R-seq is significantly sparser than that from DEFND-seq.

We thank the reviewer for the positive comments. We have now revised the discussion to include advantages and disadvantages of HIPSD&R-seq as compared to DEFND-seq.

**Reviewer #2:** Otonicar et al. have significantly improved the manuscript with the following components: 1) additional analysis of CNV calling accuracy and robustness; 2) additional analysis of transcriptome; and most importantly, 3) benchmark comparison against a similar method DEFEND and demonstrated the better performance. These additional analysis results highlighted the power of HIPSD&R in identifying rare CNV-defined populations in cell mixtures of different origins. I would like to suggest two changes in wording before fully acceptance. And additional review is not needed.

1. The authors showed the minimal TSS enrichment score and the lack of nucleosome periodicity patterns in HIPSD dataset, which suggested that the nucleosomes were efficiently depleted so that their impact on CNV inference was minimal. By quantifying the correlation with bulk CNV, the authors further concluded that the HIPSD&R DANN outperformed scRNA-seq or scATAC-seq. But the Pearson’s correlation coefficient compared to bulk is around 0.6 at 1MB resolution. This correlation should warrant robust clustering analysis, but probably not high enough to be stated as “accurate CNV calling”.

I will probably state it as robust CNV calling.

Consistent with this thought, the results of “metacell” CNV does have some deviations from bulk CNV calling in Supplementary Fig. 9, as mentioned in my original comments. I guess that it indicates that there are still some regional biases at 1MB resolution. Just out of curiosity, will these deviations be minimized if enlarging the binning windows, for both single cells and metacells?

We now refer to the CNV calling as robust instead of accurate in the revised text.

Regarding the deviations from bulk CNV calling:

It is likely that deviations compared to bulk would be further minimized by increasing the bin size. However, we would like to emphasize that the parameters of HMMCopy have to be optimized for a specific bin size. In our case, we focused on 100kb for metacells and 1Mb for single cells.

We have now performed additional analyses presented in revised Supplementary Figure 6 on the size resolution. These new analyses further support the robust CNV calling of HIPSD&R-seq data down to 100kb bin size.

2. I would like to suggest that the authors to provide more detailed descriptions when referring to Supplementary Figs. 1-3. and maybe provide a summary of the results in the main text.

We now provide more detailed descriptions when referring to Supplementary figures 1-3 as well as a summary in the main text.

In addition, we would like to mention that the correct passage number for patient LFS041 HIPSD-seq is passage 62 and not passage 63 as initially stated in the mansucript, we have now corrected this in Table S1 and in the revised text.
